# Estimation of COVID-19 mRNA Vaccine Effectiveness and COVID-19 Illness and Severity by Vaccination Status During Omicron BA.4 and BA.5 Sublineage Periods

**DOI:** 10.1001/jamanetworkopen.2023.2598

**Published:** 2023-03-15

**Authors:** Ruth Link-Gelles, Matthew E. Levy, Karthik Natarajan, Sarah E. Reese, Allison L. Naleway, Shaun J. Grannis, Nicola P. Klein, Malini B. DeSilva, Toan C. Ong, Manjusha Gaglani, Emily Hartmann, Monica Dickerson, Edward Stenehjem, Anupam B. Kharbanda, Jungmi Han, Talia L. Spark, Stephanie A. Irving, Brian E. Dixon, Ousseny Zerbo, Charlene E. McEvoy, Suchitra Rao, Chandni Raiyani, Chantel Sloan-Aagard, Palak Patel, Kristin Dascomb, Anne-Catrin Uhlemann, Margaret M. Dunne, William F. Fadel, Ned Lewis, Michelle A. Barron, Kempapura Murthy, Juan Nanez, Eric P. Griggs, Nancy Grisel, Medini K. Annavajhala, Akintunde Akinseye, Nimish R. Valvi, Kristin Goddard, Mufaddal Mamawala, Julie Arndorfer, Duck-Hye Yang, Peter J. Embí, Bruce Fireman, Sarah W. Ball, Mark W. Tenforde

**Affiliations:** 1 Centers for Disease Control and Prevention COVID-19 Response Team, Atlanta, Georgia; 2 Westat, Rockville, Maryland; 3 Department of Biomedical Informatics, Columbia University Irving Medical Center, New York, New York; 4 New York–Presbyterian Hospital, New York, New York; 5 Kaiser Permanente Center for Health Research, Portland, Oregon; 6 Center for Biomedical Informatics, Regenstrief Institute, Indianapolis, Indiana; 7 School of Medicine, Indiana University, Indianapolis; 8 Kaiser Permanente Vaccine Study Center, Kaiser Permanente Northern California Division of Research, Oakland; 9 HealthPartners Institute, Minneapolis, Minnesota; 10 Department of Pediatrics, University of Colorado Anschutz Medical Campus, Aurora; 11 Baylor Scott and White Health, Temple, Texas; 12 Texas A&M University College of Medicine, Temple; 13 Paso del Norte Health Information Exchange, El Paso, Texas; 14 Division of Infectious Diseases and Clinical Epidemiology, Intermountain Healthcare, Salt Lake City, Utah; 15 Children’s Minnesota, Minneapolis; 16 Fairbanks School of Public Health, Indiana University, Indianapolis; 17 Department of Public Health, Brigham Young University, Provo, Utah; 18 Department of Internal Medicine, Division of Infectious Disease, Columbia University Irving Medical Center, New York, New York; 19 Vanderbilt University Medical Center, Nashville, Tennessee

## Abstract

**Importance:**

Recent SARS-CoV-2 Omicron variant sublineages, including BA.4 and BA.5, may be associated with greater immune evasion and less protection against COVID-19 after vaccination.

**Objectives:**

To evaluate the estimated vaccine effectiveness (VE) of 2, 3, or 4 doses of COVID-19 mRNA vaccination among immunocompetent adults during a period of BA.4 or BA.5 predominant circulation; and to evaluate the relative severity of COVID-19 in hospitalized patients across Omicron BA.1, BA.2 or BA.2.12.1, and BA.4 or BA.5 sublineage periods.

**Design, Setting, and Participants:**

This test-negative case-control study was conducted in 10 states with data from emergency department (ED) and urgent care (UC) encounters and hospitalizations from December 16, 2021, to August 20, 2022. Participants included adults with COVID-19–like illness and molecular testing for SARS-CoV-2. Data were analyzed from August 2 to September 21, 2022.

**Exposures:**

mRNA COVID-19 vaccination.

**Main Outcomes and Measures:**

The outcomes of interest were COVID-19 ED or UC encounters, hospitalizations, and admission to the intensive care unit (ICU) or in-hospital death. VE associated with protection against medically attended COVID-19 was estimated, stratified by care setting and vaccine doses (2, 3, or 4 doses vs 0 doses as the reference group). Among hospitalized patients with COVID-19, demographic and clinical characteristics and in-hospital outcomes were compared across sublineage periods.

**Results:**

During the BA.4 and BA.5 predominant period, there were 82 229 eligible ED and UC encounters among patients with COVID-19–like illness (median [IQR] age, 51 [33-70] years; 49 682 [60.4%] female patients), and 19 114 patients (23.2%) had test results positive for SARS-CoV-2; among 21 007 hospitalized patients (median [IQR] age, 71 [58-81] years; 11 209 [53.4%] female patients), 3583 (17.1 %) had test results positive for SARS-CoV-2. Estimated VE against hospitalization was 25% (95% CI, 17%-32%) for receipt of 2 vaccine doses at 150 days or more after receipt, 68% (95% CI, 50%-80%) for a third dose 7 to 119 days after receipt, and 36% (95% CI, 29%-42%) for a third dose 120 days or more (median [IQR], 235 [204-262] days) after receipt. Among patients aged 65 years or older who had received a fourth vaccine dose, VE was 66% (95% CI, 53%-75%) at 7 to 59 days after vaccination and 57% (95% CI, 44%-66%) at 60 days or more (median [IQR], 88 [75-105] days) after vaccination. Among hospitalized patients with COVID-19, ICU admission or in-hospital death occurred in 21.4% of patients during the BA.1 period vs 14.7% during the BA.4 and BA.5 period (standardized mean difference: 0.17).

**Conclusions and Relevance:**

In this case-control study of COVID-19 vaccines and illness, VE associated with protection against medically attended COVID-19 illness was lower with increasing time since last dose; estimated VE was higher after receipt of 1 or 2 booster doses compared with a primary series alone.

## Key Points

**Question:**

What is the estimated vaccine effectiveness (VE) associated with first-generation COVID-19 mRNA vaccines against medically attended COVID-19 during Omicron BA.4 and BA.5 sublineage predominance?

**Findings:**

This case-control study included 82 229 emergency department or urgent care encounters and 21 007 hospitalizations for COVID-19–like illness. Among hospitalized patients, estimated 3-dose VE was 68% for those with the third dose 7 to 119 days prior, but was lower by 120 days or longer after vaccination (VE, 36%).

**Meaning:**

These findings suggest that first-generation COVID-19 mRNA vaccines were associated with protection against COVID-19 during the Omicron BA.4/BA.5 sublineage-predominant periods but protection declined over time.

## Teaser

This case control study evaluates the estimated vaccine effectiveness associated with 2, 3, or 4 doses of COVID-19 mRNA vaccination for protection against emergency department or urgent care encounters and against hospitalization among immunocompetent adults during a periods of Omicron BA.4 and BA.5 sublineage-predominant circulation.

## Introduction

COVID-19 vaccines are estimated to have prevented tens of thousands of COVID-19–associated hospitalizations and deaths in the US.^1^ However, over the course of the pandemic new SARS-CoV-2 variants have continued to emerge and evade vaccine-induced immunity.^2^ Following a Delta variant–predominant period, the Omicron BA.1 sublineage became predominant in the United States by December 2021. Compared with earlier SARS-CoV-2 variants, BA.1 demonstrated increased transmissibility and immune evasion with a reduction in vaccine effectiveness (VE) offset by COVID-19 vaccine booster doses.^3,4^ Omicron has since diversified into additional sublineages, including several with greater immune escape potential compared with BA.1 (eg, BA.2.12.1, BA.4, and BA.5).^5,6^ BA.4 and BA.5 sublineages, which share a common spike protein, became the predominant sublineages in the US in June 2022.^7^

As new variants emerge, ongoing monitoring of VE is critical for informing public health strategies and policies. COVID-19 VE estimation has become increasingly complex as additional vaccine booster doses are authorized, vaccine-induced protection wanes over time, new variants or subvariants emerge, and most of the US population has experienced previous infection (57%-94%, depending on source).^8,9,10,11,12^ In November 2021, all adults were recommended to receive a third (first booster) vaccine dose after a 2-dose primary series of mRNA vaccine; in March 2022, adults aged 50 years or older were recommended to receive a fourth dose (second booster) at least 4 months after dose 3.^13^ In September 2022, bivalent mRNA vaccine booster doses for all individuals aged 12 years or older (Pfizer-BioNTech) and adults aged 18 years or older (Moderna) were recommended at least 2 months after completing a primary series or receiving a third dose.^14^ Like first-generation vaccines, bivalent vaccines contain an mRNA component targeting the ancestral virus in addition to a new component targeting the BA.4 and BA.5 spike protein.

The objectives for this analysis were (1) to estimate the VE associated with first-generation mRNA vaccines (BNT162b2 from Pfizer-BioNTech and mRNA-1273 from Moderna) against medical encounters for COVID-19–related illness during a period of BA.4 and BA.5 Omicron sublineage predominance among adults without immunocompromising conditions and (2) to describe the characteristics and illness severity among hospitalized patients with COVID-19 during the Omicron BA.4- and BA.5-predominant period compared with prior Omicron sublineage periods (BA.1 and BA.2 or BA.2.12.1). Understanding changes in the epidemiology of COVID-19 and VE will inform interpretation of VE studies for recently authorized bivalent vaccines.

## Methods

This case-control study was reviewed and approved by the institutional review boards at participating sites and under a reliance agreement between the Centers for Disease Control and Prevention (CDC) and the Westat institutional review board. This activity was reviewed by CDC and was conducted consistent with applicable federal law and CDC policy (eg, 45 CFR part 46.102(l)(2), 21 CFR part 56; 42 USC §241(d); 5 USC §552a; 44 USC §3501). This study presented minimal risk to participants because there was no interaction or intervention with patients; therefore, a waiver of informed consent was granted. This study followed the Strengthening the Reporting of Observational Studies in Epidemiology (STROBE) reporting guideline.

### Design and Setting

The VISION Network is a multistate collaboration between the CDC and health care systems with integrated medical, laboratory, and vaccination records. The VISION Network performs serial assessments of COVID-19 VE in emergency department (ED), urgent care (UC), and hospital settings using the test-negative case-control design.^15^ Nine VISION Network health care systems in 10 states contributed data for this analysis (eTable 1 in Supplement 1), including 268 hospitals, 292 EDs, and 140 UC clinics.

To calculate estimated VE, we assessed ED and UC encounters and hospitalizations with 1 or more discharge codes related to COVID-19–like illness (*International Classification of Diseases, Ninth Revision* [*ICD-9*] and *International Statistical Classification of Diseases and Related Health Problems, Tenth Revision* [*ICD-10*]) (eTable 2 in Supplement 1) and a molecular test (primarily reverse transcription–polymerase chain reaction [RT-PCR] assay) for SARS-CoV-2 performed within 14 days before or up to less than 72 hours after the encounter during a BA.4- and BA.5-predominant period prior to authorization of bivalent booster doses, June 19 to August 20, 2022. Site-specific start dates were defined from local sequencing data when the combined prevalence of BA.4 and BA.5 was at least 50% and continued through the end of the study period (August 20, 2022). COVID-19 cases included patients with at least 1 COVID-19–like illness code and a positive SARS-CoV-2 molecular test result; controls included patients with at least 1 COVID-19–like illness code and a negative SARS-CoV-2 molecular test result.

For the comparison of severity by Omicron sublineage period, we included hospitalized patients with COVID-19 during BA.1 predominance (encounters December 2021 to March 2022), combined BA.2 and BA.2.12.1 (encounters March to June 2022), and BA.4 and BA.5 (encounters June to August 2022) sublineage-predominant periods. Baseline characteristics of hospitalized patients with COVID-19, including demographics, underlying medical conditions, prior vaccination and prior infection histories, and in-hospital outcomes (hospital length of stay, intensive care unit [ICU] admission, invasive mechanical ventilation, and in-hospital death within 28 days of admission), were obtained through electronic medical records and compared by period.

### Participants

We included adults aged 18 years or older with a medical encounter related to COVID-19–like illness and SARS-CoV-2 molecular testing. COVID-19–like illness encounters include *ICD-9* or *ICD-10* codes for acute respiratory clinical diagnoses (eg, pneumonia, respiratory failure) or COVID-19–related signs or symptoms (eg, shortness of breath, cough, fever) during an UC or ED visit or a hospital admission with at least 24 hours’ duration. Repeat ED or UC visits within a 24-hour period or multiple hospital admissions that occurred within a 30-day period (from prior discharge) were combined into a single event with the earliest date used as the index date to determine vaccination status. One individual could contribute more than 1 event during the analysis period. Information on patients’ baseline characteristics, including demographic characteristics, such as race (Black, White, and other [eg, Asian, American Indian or Alaska Native, Native Hawaiian or other Pacific Islander, multiracial, and other not listed]) and ethnicity (Hispanic and non-Hispanic), underlying medical conditions, and prior SARS-CoV-2 testing results, was obtained through the health systems’ electronic medical records and *ICD-9* and *ICD-10* codes. Only completed hospitalizations (ie, hospital events in which a patient was discharged or died) were included in this analysis.

### Classification of Vaccination Status

COVID-19 vaccination status was ascertained through state or local immunization information systems, electronic medical records, and claims data. Only mRNA vaccines were considered in this analysis. Vaccination status was assigned using doses received prior to a medical encounter index date, defined as either the date of collection of a respiratory specimen associated with the most recent positive or negative SARS-CoV-2 test result before the medical visit or the date of the medical visit (if testing occurred only after the admission or visit date). Patients were considered unvaccinated if no mRNA vaccine doses were received before the index date; vaccinated with a primary series if 2 doses were received with the second dose at least 14 days before the index date; vaccinated with a first booster if 3 doses were received with the third dose at least 7 days before the index date; or, among patients aged at least 50 years, vaccinated with second booster if a fourth dose was received at least 7 days before the index date. Patients were excluded if they received a third or fourth dose before recommended for immunocompetent adults or received a dose with a shorter interval than recommended (ie, less than 5 months between second and third dose or less than 4 months between third and fourth dose). Patients were excluded if they received only 1 mRNA vaccine dose, received a non-mRNA vaccine (eg, viral vector), or had a likely immunocompromising condition, as previously defined.^16^

### Statistical Analysis

The association of symptomatic laboratory-confirmed SARS-CoV-2 infection at an ED or UC encounter or hospitalization with vaccination status was estimated among individuals with COVID-19-like illness using multivariable logistic regression. Odds ratios (ORs) were calculated by comparing the odds of prior receipt of 2, 3, or 4 vaccine doses vs unvaccinated status (reference group) between patients with confirmed SARS-CoV-2 infection vs those with negative results from SARS-CoV-2 testing. VE was estimated as (1 – OR) × 100% for the ORs for 2, 3, and 4 doses vs unvaccinated against COVID-19–related ED or UC encounters or hospitalizations. To evaluate VE against more severe COVID-19, a further analysis was performed among hospitalized patients with COVID-19 comparing only patients who were admitted to the ICU or experienced in-hospital death with hospitalized controls.^17^ Two-dose, 3-dose, and 4-dose VE estimates were further stratified by time periods since most recent vaccination dose (ie, 2-dose, 14-149 days; 2-dose, ≥150 days; 3-dose, 7-119 days; 3 dose, ≥120 days; 4-dose, 7-59 days; and 4-dose, ≥60 days).

In addition to estimating absolute VE (ie, VE for receipt of vaccine compared with unvaccinated), ORs were also calculated to estimate relative VE (rVE) to estimate the incremental benefit associated with receiving an additional vaccine dose when recommended. rVE was estimated by comparing individuals who had recently received 1 or 2 booster doses with those who were eligible for but had not received the respective booster dose, ie, persons who had received a third dose within the last 7 to 119 days vs persons who had received a second dose 150 or more days prior and persons who had received a fourth dose within the last 7 to 119 days vs persons who had received a third dose 120 or more days prior.

VEs were estimated separately among ED or UC encounters and hospitalizations for any combination of mRNA vaccine products and stratified by age group (18-49, 50-64, and ≥65 years). Two additional sensitivity analyses were conducted: stratified by vaccine product received and restricted to patients without a prior SARS-CoV-2 infection documented in electronic medical records.

All models included covariates for age, geographic region, calendar time, and level of local SARS-CoV-2 circulation (7-day moving mean of percentage of RT-PCR tests that were positive for SARS-CoV-2 within the medical facility’s geographic region). Age, calendar time, and SARS-CoV-2 circulation level covariates were specified as natural cubic spline functions with knots at quartiles. For models estimating the absolute OR, cases and controls were propensity score–weighted using the inverse probability of being vaccinated (if vaccinated) or unvaccinated (if not vaccinated). For models estimating relative ORs, a similar method was used based on patients’ inverse propensity to be 3-dose vs 2-dose vaccinated or 4-dose vs 3-dose vaccinated. Generalized boosted regression trees were used to estimate the propensity score for being vaccinated based on demographics, underlying medical conditions, and facility characteristics. Separate weights were calculated for each model and were truncated at the 99th percentile of the distribution of weights. After weighting, an absolute standardized mean difference (SMD) of 0.20 or less was taken to indicate a negligible difference in distributions of covariates by vaccination status. Any covariates with an SMD greater than 0.20 after weighting were also included in the model in addition to the a priori variables for the respective OR estimate to minimize residual confounding (eTable 3 in Supplement 1). Two-sided 95% CIs were calculated for each VE estimate, with 95% CIs that excluded 0 considered statistically significant. Nonoverlapping CIs were interpreted as statistically different VEs.

To describe outcomes of patients hospitalized with COVID-19 during the BA.4- and BA.5-predominant period compared with earlier sublineage periods, we restricted to hospitalized patients during BA.1, BA.2 and BA.2.12.1, and BA.4 and BA.5 periods who met aforementioned inclusion criteria. Baseline demographic, clinical, and vaccination characteristics and in-hospital outcomes were compared between patients with COVID-19 during BA.4 and BA.5 periods and those during other sublineage periods using SMDs.

Analyses were performed using R software version 4.1.2 (R Project for Statistical Computing) and SAS software version 9.4 (SAS Institute). Detailed methods are included in the eAppendix, eTable 1, and eTable 2 in Supplement 1. Data were analyzed from August 2 to September 21, 2022.

## Results

### Participants Included in Analysis to Estimate VE

Among 253 367 ED and UC encounters during the BA.4 and BA.5 period, there were 82 229 eligible ED or UC encounters related to COVID-19–like illness (median [IQR] patient age, 51 [33-70] years; 49 682 [60.4%] female patients) (eFigure 1 in Supplement 1); 19 114 encounters (23.2%) included a positive SARS-CoV-2 test result. Among eligible encounters, 12 872 (15.7%) were Hispanic patients, 10 300 (12.5%) were non-Hispanic Black patients, and 48 753 (59.3%) were non-Hispanic White patients (Table 1). A total of 171 138 ED and UC encounters were excluded from analysis.

**Table 1.** Characteristics of ED or UC Encounters Included in Analysis

Characteristic	SARS-CoV-2 status			mRNA COVID-19 vaccination status								Overall, No. (%)
	No. (%)^a^		SMD	No. (%)^a^							SMD	
	Cases (positive)	Controls (negative)		Unvaccinated	2 doses, 14-149 d earlier	2 doses, ≥150 d earlier	3 doses, 7-119 d earlier	3 doses, ≥120 d earlier	4 doses, 7-59 d earlier	4 doses, ≥60 d earlier		
All ED or UC encounters^b^	19 114 (23.2)	63 115 (76.8)	NA	29 365 (35.7)	652 (0.8)	19 594 (23.8)	1539 (1.9)	23 417 (28.5)	2491 (3.0)	5171 (6.3)	NA	82 229 (100)
Site												
Baylor Scott and White Health	5344 (33.2)	10 744 (66.8)	0.37	8440 (52.5)	86 (0.5)	4679 (29.1)	150 (0.9)	2424 (15.1)	110 (0.7)	199 (1.2)	1.02	16 088 (19.6)
Columbia University^c^	294 (13.6)	1865 (86.4)		840 (38.9)	22 (1.0)	622 (28.8)	48 (2.2)	553 (25.6)	17 (0.8)	57 (2.6)		2159 (2.6)
HealthPartners^c^	2122 (19.1)	9000 (80.9)		2752 (24.7)	115 (1.0)	2304 (20.7)	282 (2.5)	4007 (36.0)	541 (4.9)	1121 (10.1)		11 122 (13.5)
Intermountain Healthcare	3852 (27.3)	10 273 (72.7)		4671 (33.1)	162 (1.1)	3662 (25.9)	308 (2.2)	4357 (30.8)	356 (2.5)	609 (4.3)		14 125 (17.2)
KPNC	2543 (15.7)	13 639 (84.3)		2128 (13.2)	131 (0.8)	3109 (19.2)	542 (3.3)	6806 (42.1)	1089 (6.7)	2377 (14.7)		16 182 (19.7)
KPCHR	1124 (22.4)	3897 (77.6)		1280 (25.5)	35 (0.7)	980 (19.5)	108 (2.2)	1814 (36.1)	229 (4.6)	575 (11.5)		5021 (6.1)
PHIX^c^	77 (16.1)	401 (83.9)		201 (42.1)	1 (0.2)	135 (28.2)	14 (2.9)	106 (22.2)	6 (1.3)	15 (3.1)		478 (0.6)
Regenstrief Institute	2547 (19.7)	10 365 (80.3)		6947 (53.8)	87 (0.7)	2958 (22.9)	44 (0.3)	2642 (20.5)	85 (0.7)	149 (1.2)		12 912 (15.7)
University of Colorado	1211 (29.2)	2931 (70.8)		2106 (50.8)	13 (0.3)	1145 (27.6)	43 (1.0)	708 (17.1)	58 (1.4)	69 (1.7)		4142 (5.0)
Age group, y												
18-49	9474 (24.2)	29 647 (75.8)	0.06	19 177 (49.0)	358 (0.9)	10 409 (26.6)	619 (1.6)	8558 (21.9)	0	0	0.99	39 121 (47.6)
50-64	3795 (23.4)	12 402 (76.6)		5117 (31.6)	127 (0.8)	4080 (25.2)	360 (2.2)	4978 (30.7)	609 (3.8)	926 (5.7)		16 197 (19.7)
65-74	2542 (22.0)	9019 (78.0)		2488 (21.5)	78 (0.7)	2321 (20.1)	222 (1.9)	4148 (35.9)	719 (6.2)	1585 (13.7)		11 561 (14.1)
75-84	2133 (21.6)	7721 (78.4)		1737 (17.6)	59 (0.6)	1806 (18.3)	227 (2.3)	3658 (37.1)	704 (7.1)	1663 (16.9)		9854 (12.0)
≥85	1170 (21.3)	4326 (78.7)		846 (15.4)	30 (0.5)	978 (17.8)	111 (2.0)	2075 (37.8)	459 (8.4)	997 (18.1)		5496 (6.7)
Sex												
Male	7728 (23.7)	24 819 (76.3)	0.02	12 239 (37.6)	231 (0.7)	7230 (22.2)	595 (1.8)	8880 (27.3)	1049 (3.2)	2323 (7.1)	0.08	32 547 (39.6)
Female	11 386 (22.9)	38 296 (77.1)		17 126 (34.5)	421 (0.8)	12 364 (24.9)	944 (1.9)	14 537 (29.3)	1442 (2.9)	2848 (5.7)		49 682 (60.4)
Race and ethnicity												
Hispanic	3007 (23.4)	9865 (76.6)	0.08	4838 (37.6)	122 (0.9)	3513 (27.3)	261 (2.0)	3373 (26.2)	286 (2.2)	479 (3.7)	0.34	12 872 (15.7)
Non-Hispanic												
Black	2713 (26.3)	7587 (73.7)		4959 (48.1)	119 (1.2)	2622 (25.5)	236 (2.3)	1948 (18.9)	158 (1.5)	258 (2.5)		10 300 (12.5)
White	10 908 (22.4)	37 845 (77.6)		16 064 (32.9)	329 (0.7)	11 160 (22.9)	847 (1.7)	14 779 (30.3)	1762 (3.6)	3812 (7.8)		48 753 (59.3)
Other^d^	1620 (23.1)	5398 (76.9)		1812 (25.8)	60 (0.9)	1500 (21.4)	164 (2.3)	2641 (37.6)	264 (3.8)	577 (8.2)		7018 (8.5)
Unknown race and ethnicity	866 (26.4)	2420 (73.6)		1692 (51.5)	22 (0.7)	799 (24.3)	31 (0.9)	676 (20.6)	21 (0.6)	45 (1.4)		3286 (4.0)
Documented prior SARS-CoV-2 infection												
Yes	2360 (15.9)	12 469 (84.1)	0.20	5779 (39.0)	186 (1.3)	4196 (28.3)	371 (2.5)	3555 (24.0)	280 (1.9)	462 (3.1)	0.17	14 829 (18.0)
No	16 754 (24.9)	50 646 (75.1)		23 586 (35.0)	466 (0.7)	15 398 (22.8)	1168 (1.7)	19 862 (29.5)	2211 (3.3)	4709 (7.0)		67 400 (82.0)
SARS-CoV-2 status												
Positive case	19 114 (100)	NA	NA	8401 (44.0)	96 (0.5)	4436 (23.2)	175 (0.9)	4909 (25.7)	292 (1.5)	805 (4.2)	0.31	19 114 (23.2)
Negative control	NA	63 115 (100)		20 964 (33.2)	556 (0.9)	15 158 (24.0)	1364 (2.2)	18 508 (29.3)	2199 (3.5)	4366 (6.9)		63 115 (76.8)
mRNA COVID-19 vaccination status												
Unvaccinated	8401 (28.6)	20 964 (71.4)	0.28	29 365 (100)	NA	NA	NA	NA	NA	NA	NA	29 365 (35.7)
2 Doses, 14-149 d earlier	96 (14.7)	556 (85.3)		NA	652 (100)	NA	NA	NA	NA	NA	NA	652 (0.8)
2 Doses, ≥150 d earlier	4436 (22.6)	15 158 (77.4)		NA	NA	19 594 (100)	NA	NA	NA	NA	NA	19 594 (23.8)
3 Doses, 7-119 d earlier	175 (11.4)	1364 (88.6)		NA	NA	NA	1539 (100)	NA	NA	NA	NA	1539 (1.9)
3 Doses, ≥120 d earlier	4909 (21.0)	18 508 (79.0)		NA	NA	NA	NA	23 417 (100)	NA	NA	NA	23 417 (28.5)
4 Doses, 7-59 d earlier	292 (11.7)	2199 (88.3)		NA	NA	NA	NA	NA	2491 (100)	NA	NA	2491 (3.0)
4 Doses, ≥60 d earlier	805 (15.6)	4366 (84.4)		NA	NA	NA	NA	NA	NA	5171 (100)	NA	5171 (6.3)
≥1 Chronic respiratory condition^e^												
Yes	2174 (18.6)	9523 (81.4)	0.11	3778 (32.3)	95 (0.8)	2926 (25.0)	259 (2.2)	3457 (29.6)	376 (3.2)	806 (6.9)	0.07	11 697 (14.2)
No	16 940 (24.0)	53 592 (76.0)		25 587 (36.3)	557 (0.8)	16 668 (23.6)	1280 (1.8)	19 960 (28.3)	2115 (3.0)	4365 (6.2)		70 532 (85.8)
≥1 Chronic nonrespiratory condition^f^												
Yes	3948 (18.5)	17 411 (81.5)	0.16	6722 (31.5)	158 (0.7)	5226 (24.5)	498 (2.3)	6278 (29.4)	800 (3.7)	1677 (7.9)	0.14	21 359 (26.0)
No	15 166 (24.9)	45 704 (75.1)		22 643 (37.2)	494 (0.8)	14 368 (23.6)	1041 (1.7)	17 139 (28.2)	1691 (2.8)	3494 (5.7)		60 870 (74.0)

Abbreviations: ED, emergency department; KPCHR, Kaiser Permanente Center for Health Research; KPNC, Kaiser Permanente Northern California; NA, not applicable; PHIX, Paso del Norte Health Information Exchange; SMD, standardized mean or proportion difference; UC, urgent care.

^a^ Percentages are given by row.

^b^ Medical encounters were included during dates of estimated at least 50% Omicron BA.4/BA.5 sublineage predominance, reported in eTable 1 in Supplement 1.

^c^ ED data from Columbia University, HealthPartners, and PHIX exclude encounters that were transferred to an inpatient setting.

^d^ Other race includes American Indian or Alaska Native, Asian, Native Hawaiian or other Pacific Islander, other, and multiple races.

^e^ Chronic respiratory condition was defined as the presence of discharge code for asthma, chronic obstructive pulmonary disease, or other lung disease.

^f^ Chronic nonrespiratory condition was defined as the presence of discharge code for heart failure, ischemic heart disease, hypertension, other heart disease, stroke, other cerebrovascular disease, diabetes type 1 or 2, other diabetes, metabolic disease, clinical obesity, clinically underweight, kidney disease, liver disease, blood disorder, immunosuppression, organ transplant, cancer, dementia, neurologic disorder, musculoskeletal disorder, or Down syndrome.

Among included ED and UC encounters, patients with COVID-19 were less likely to have received at least 1 booster dose compared with controls (6181 patients [32.3%] vs 26 437 patients [41.9%]) but were similar in age to controls (median [IQR] age, 50 [33-69] years vs 52 [30-70] years) (Table 1). Among patients aged at least 50 years, 1097 patients with COVID-19 (11.4%) with ED or UC encounters had received a second booster (fourth dose) compared with 6565 patients without COVID-19 (19.6%).

**Figure 1.**
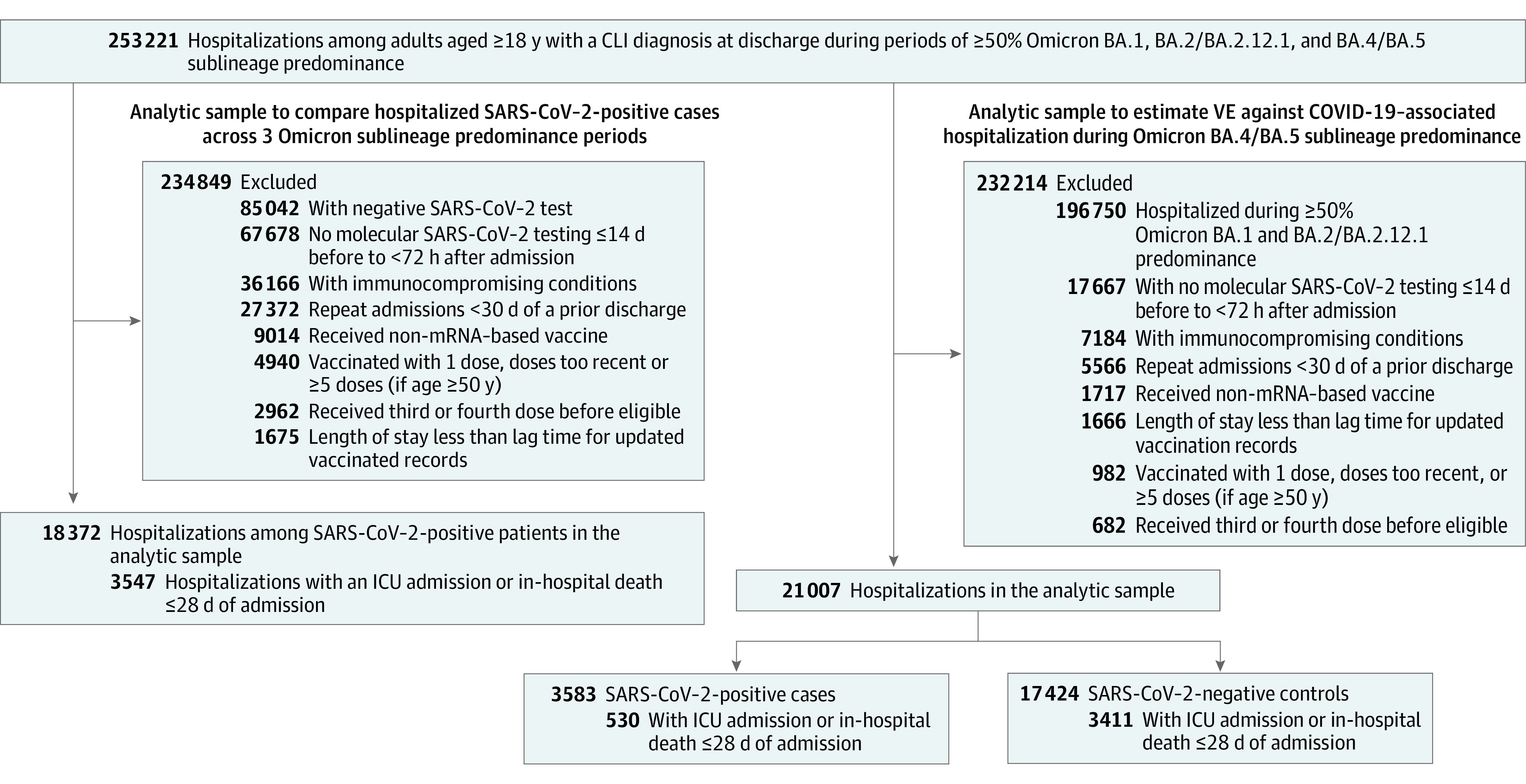
Flowchart for the Selection of Hospitalizations CLI indicates COVID-19–like illness; ICU, intensive care unit; VE, vaccine effectiveness.

Among 56 471 hospitalizations during the BA.4 and BA.5 period, there were 21 007 eligible hospitalizations (median [IQR] patient age, 71 [58-81] years; 11 209 [53.4%] female patients) included in the analysis (Figure 1). A total of 3583 patients (17.1%) had a positive SARS-CoV-2 test result. There were 2370 Hispanic patients (11.3%), 2362 non-Hispanic Black patients (11.2%), and 13 620 non-Hispanic White patients (64.8%) (Table 2). A total of 35 464 hospitalizations were excluded from analysis.

**Table 2.** Characteristics of Hospitalizations Included in Analysis

Characteristic	SARS-CoV-2 status			mRNA COVID-19 vaccination status								Overall, No. (%)
	No. (%)^a^		SMD	No. (%)^a^							SMD	
	Cases (positive)	Controls (negative)		Unvaccinated	2 doses, 14-149 d earlier	2 doses, ≥150 d earlier	3 doses, 7-119 d earlier	3 doses, ≥120 d earlier	4 doses, 7-59 d earlier	4 doses, ≥60 d earlier		
All hospitalizations^b^	3583 (17.1)	17 424 (82.9)	NA	6337 (30.2)	141 (0.7)	4845 (23.1)	429 (2.0)	6656 (31.7)	878 (4.2)	1721 (8.2)	NA	21 007 (100)
Site												
Baylor Scott and White Health	875 (19.6)	3596 (80.4)	0.18	1927 (43.1)	19 (0.4)	1435 (32.1)	58 (1.3)	889 (19.9)	43 (1.0)	100 (2.2)	1.01	4471 (21.3)
Columbia University	119 (14.2)	718 (85.8)		264 (31.5)	12 (1.4)	190 (22.7)	26 (3.1)	283 (33.8)	24 (2.9)	38 (4.5)		837 (4.0)
HealthPartners	151 (11.2)	1194 (88.8)		272 (20.2)	13 (1.0)	225 (16.7)	51 (3.8)	484 (36.0)	104 (7.7)	196 (14.6)		1345 (6.4)
Intermountain Healthcare	380 (21.2)	1412 (78.8)		506 (28.2)	17 (0.9)	390 (21.8)	60 (3.3)	619 (34.5)	79 (4.4)	121 (6.8)		1792 (8.5)
KPNC	890 (17.1)	4306 (82.9)		552 (10.6)	41 (0.8)	805 (15.5)	169 (3.3)	2157 (41.5)	468 (9.0)	1004 (19.3)		5196 (24.7)
KPCHR	103 (13.0)	691 (87.0)		227 (28.6)	5 (0.6)	120 (15.1)	13 (1.6)	254 (32.0)	55 (6.9)	120 (15.1)		794 (3.8)
PHIX	14 (20.0)	56 (80.0)		26 (37.1)	0	17 (24.3)	0	20 (28.6)	1 (1.4)	6 (8.6)		70 (0.3)
Regenstrief Institute	892 (16.1)	4652 (83.9)		2166 (39.1)	27 (0.5)	1393 (25.1)	30 (0.5)	1742 (31.4)	84 (1.5)	102 (1.8)		5544 (26.4)
University of Colorado	159 (16.6)	799 (83.4)		397 (41.4)	7 (0.7)	270 (28.2)	22 (2.3)	208 (21.7)	20 (2.1)	34 (3.5)		958 (4.6)
Age group, y												
18-49	433 (12.6)	2995 (87.4)	0.25	1749 (51.0)	34 (1.0)	887 (25.9)	61 (1.8)	697 (20.3)	0	0	0.62	3428 (16.3)
50-64	599 (14.1)	3663 (85.9)		1617 (37.9)	30 (0.7)	1130 (26.5)	79 (1.9)	1121 (26.3)	113 (2.7)	172 (4.0)		4262 (20.3)
65-74	734 (15.6)	3964 (84.4)		1243 (26.5)	30 (0.6)	1062 (22.6)	96 (2.0)	1587 (33.8)	241 (5.1)	439 (9.3)		4698 (22.4)
75-84	1025 (20.1)	4082 (79.9)		1100 (21.5)	29 (0.6)	1067 (20.9)	108 (2.1)	1895 (37.1)	302 (5.9)	606 (11.9)		5107 (24.3)
≥85	792 (22.6)	2720 (77.4)		628 (17.9)	18 (0.5)	699 (19.9)	85 (2.4)	1356 (38.6)	222 (6.3)	504 (14.4)		3512 (16.7)
Sex												
Male	1740 (17.8)	8058 (82.2)	0.05	3095 (31.6)	78 (0.8)	2117 (21.6)	201 (2.1)	3075 (31.4)	396 (4.0)	836 (8.5)	0.07	9798 (46.6)
Female	1843 (16.4)	9366 (83.6)		3242 (28.9)	63 (0.6)	2728 (24.3)	228 (2.0)	3581 (31.9)	482 (4.3)	885 (7.9)		11 209 (53.4)
Race and ethnicity												
Hispanic	392 (16.5)	1978 (83.5)	0.06	741 (31.3)	20 (0.8)	612 (25.8)	56 (2.4)	709 (29.9)	96 (4.1)	136 (5.7)	0.34	2370 (11.3)
Non-Hispanic												
Black	375 (15.9)	1987 (84.1)		981 (41.5)	23 (1.0)	603 (25.5)	63 (2.7)	558 (23.6)	51 (2.2)	83 (3.5)		2362 (11.2)
White	2398 (17.6)	11 222 (82.4)		3862 (28.4)	79 (0.6)	3098 (22.7)	257 (1.9)	4493 (33.0)	591 (4.3)	1240 (9.1)		13 620 (64.8)
Other^c^	281 (16.4)	1434 (83.6)		365 (21.3)	18 (1.0)	306 (17.8)	42 (2.4)	622 (36.3)	121 (7.1)	241 (14.1)		1715 (8.2)
Unknown race and ethnicity	137 (14.6)	803 (85.4)		388 (41.3)	1 (0.1)	226 (24.0)	11 (1.2)	274 (29.1)	19 (2.0)	21 (2.2)		940 (4.5)
Documented prior SARS-CoV-2 infection^d^												
Yes	339 (10.5)	2902 (89.5)	0.21	1047 (32.3)	31 (1.0)	919 (28.4)	101 (3.1)	921 (28.4)	104 (3.2)	118 (3.6)	0.15	3241 (15.4)
No	3244 (18.3)	14 522 (81.7)		5290 (29.8)	110 (0.6)	3926 (22.1)	328 (1.8)	5735 (32.3)	774 (4.4)	1603 (9.0)		17 766 (84.6)
SARS-CoV-2 status												
Positive case	3583 (100)	NA	NA	1266 (35.3)	18 (0.5)	824 (23.0)	33 (0.9)	1118 (31.2)	97 (2.7)	227 (6.3)	0.19	3583 (17.1)
Negative control	NA	17 424 (100)		5071 (29.1)	123 (0.7)	4021 (23.1)	396 (2.3)	5538 (31.8)	781 (4.5)	1494 (8.6)		17 424 (82.9)
mRNA COVID-19 vaccination status												
Unvaccinated	1266 (20.0)	5071 (80.0)	0.20	6337 (100)	NA	NA	NA	NA	NA	NA	NA	6337 (30.2)
2 Doses, 14-149 d earlier	18 (12.8)	123 (87.2)		NA	141 (100)	NA	NA	NA	NA	NA		141 (0.7)
2 Doses, ≥150 d earlier	824 (17.0)	4021 (83.0)		NA	NA	4845 (100)	NA	NA	NA	NA		4845 (23.1)
3 Doses, 7-119 d earlier	33 (7.7)	396 (92.3)		NA	NA	NA	429 (100)	NA	NA	NA		429 (2.0)
3 Doses, ≥120 d earlier	1118 (16.8)	5538 (83.2)		NA	NA	NA	NA	6656 (100)	NA	NA		6656 (31.7)
4 Doses, 7-59 d earlier	97 (11.0)	781 (89.0)		NA	NA	NA	NA	NA	878 (100)	NA		878 (4.2)
4 Doses, ≥60 d earlier	227 (13.2)	1494 (86.8)		NA	NA	NA	NA	NA	NA	1721 (100)		1721 (8.2)
≥1 Chronic respiratory condition^d^												
Yes	2153 (18.6)	9453 (81.4)	0.12	3312 (28.5)	83 (0.7)	2597 (22.4)	276 (2.4)	3760 (32.4)	536 (4.6)	1042 (9.0)	0.14	11 606 (55.2)
No	1430 (15.2)	7971 (84.8)		3025 (32.2)	58 (0.6)	2248 (23.9)	153 (1.6)	2896 (30.8)	342 (3.6)	679 (7.2)		9401 (44.8)
≥1 Chronic nonrespiratory condition^e^												
Yes	3165 (17.4)	15 019 (82.6)	0.06	5152 (28.3)	121 (0.7)	4166 (22.9)	399 (2.2)	5849 (32.2)	840 (4.6)	1657 (9.1)	0.29	18 184 (86.6)
No	418 (14.8)	2405 (85.2)		1185 (42.0)	20 (0.7)	679 (24.1)	30 (1.1)	807 (28.6)	38 (1.3)	64 (2.3)		2823 (13.4)
ICU admission												
Yes	465 (12.6)	3218 (87.4)	0.15	1220 (33.1)	25 (0.7)	872 (23.7)	66 (1.8)	1084 (29.4)	140 (3.8)	276 (7.5)	0.07	3683 (17.5)
No	3118 (18.0)	14 206 (82.0)		5117 (29.5)	116 (0.7)	3973 (22.9)	363 (2.1)	5572 (32.2)	738 (4.3)	1445 (8.3)		17 324 (82.5)
Receipt of invasive mechanical ventilation												
Yes	219 (12.8)	1488 (87.2)	0.10	607 (35.6)	15 (0.9)	438 (25.7)	31 (1.8)	445 (26.1)	63 (3.7)	108 (6.3)	0.44	1707 (8.1)
No	2723 (17.8)	12 610 (82.2)		4118 (26.9)	111 (0.7)	3434 (22.4)	376 (2.5)	4977 (32.5)	764 (5.0)	1553 (10.1)		15 333 (73.0)
Unknown	641 (16.2)	3326 (83.8)		1612 (40.6)	15 (0.4)	973 (24.5)	22 (0.6)	1234 (31.1)	51 (1.3)	60 (1.5)		3967 (18.9)
In-hospital death^j^												
Yes	129 (21.2)	479 (78.8)	0.05	160 (26.3)	2 (0.3)	124 (20.4)	17 (2.8)	203 (33.4)	35 (5.8)	67 (11.0)	0.06	608 (2.9)
No	3454 (16.9)	16 945 (83.1)		6177 (30.3)	139 (0.7)	4721 (23.1)	412 (2.0)	6453 (31.6)	843 (4.1)	1654 (8.1)		20 399 (97.1)

Abbreviations: ICU, intensive care unit; KPCHR, Kaiser Permanente Center for Health Research; KPNC, Kaiser Permanente Northern California; NA, not applicable; PHIX, Paso del Norte Health Information Exchange; SMD, standardized mean or proportion difference.

^a^ Percentages are given by row.

^b^ Medical encounters were included during dates of estimated at least 50% Omicron BA.4/BA.5 sublineage predominance, reported in eTable 1 in Supplement 1.

^c^ Other race includes American Indian or Alaska Native, Asian, Native Hawaiian or other Pacific Islander, other, and multiple races.

^d^ Chronic respiratory condition was defined as the presence of discharge code for asthma, chronic obstructive pulmonary disease, or other lung disease.

^e^ Chronic nonrespiratory condition was defined as the presence of discharge code for heart failure, ischemic heart disease, hypertension, other heart disease, stroke, other cerebrovascular disease, diabetes type 1 or 2, other diabetes, metabolic disease, clinical obesity, clinically underweight, kidney disease, liver disease, blood disorder, immunosuppression, organ transplant, cancer, dementia, neurologic disorder, musculoskeletal disorder, or Down syndrome.

Hospitalized patients with COVID-19 were less likely to have received at least 1 booster dose compared with controls (1475 patients [41.2%] vs 8209 patients [47.1%]) and more likely to be older (median [IQR] age, 75 [62-83] vs 70 [57-80] years) (Table 2). Among hospitalized patients aged 50 years or older, 324 patients with COVID-19 (10.3%) had received a second booster (fourth dose) compared with 2275 patients without COVID-19 (15.8%).

### Comparison of 2, 3, or 4 mRNA Vaccine Doses vs Unvaccinated

**Figure 2.**
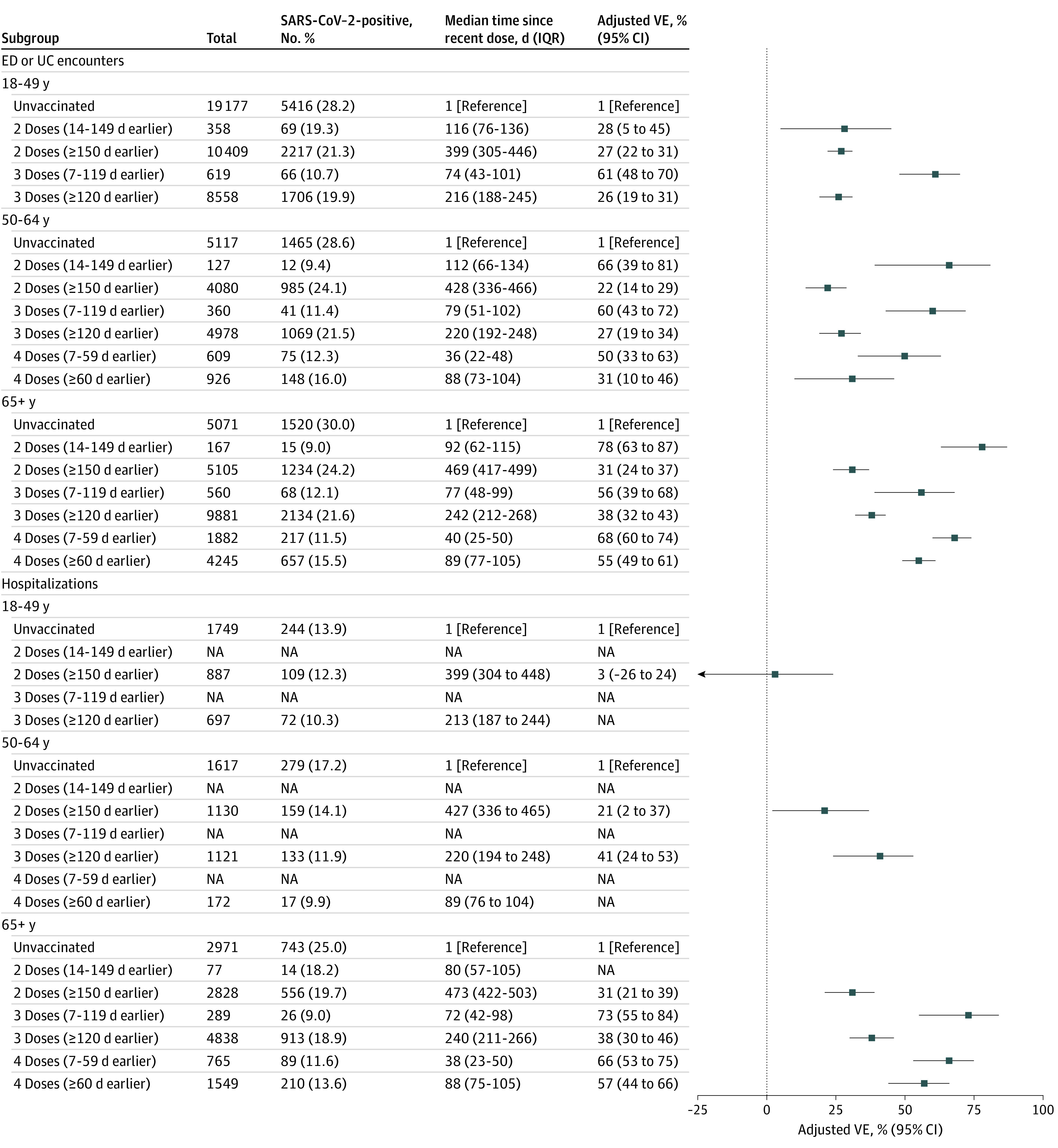
mRNA COVID-19 Vaccine Effectiveness (VE) Associated With Protection Against Laboratory-Confirmed COVID-19–Associated Emergency Department or Urgent Care Encounters and Hospitalization, by Age Group, During a Period of Omicron BA.4/BA.5 Sublineage Predominance, June 19 to August 20, 2022 VE estimates were adjusted for age, geographic region, calendar time (days since January 1, 2021), and local virus circulation (percentage of SARS-CoV-2–positive test results from testing within the counties surrounding the facility on the date of the encounter) and weighted for inverse propensity to be vaccinated or unvaccinated (calculated separately for each VE estimate). Generalized boosted regression trees were used to estimate the propensity to be vaccinated based on the following sociodemographic, facility, and medical factors: age, sex, race, ethnicity, Medicaid status, calendar date, geographic region, local SARS-CoV-2 circulation on the day of each medical visit, urban-rural classification of facility, hospital type, number of hospital beds, chronic respiratory condition, chronic nonrespiratory condition, asthma, chronic obstructive pulmonary disease, other chronic lung disease, heart failure, ischemic heart disease, hypertension, other heart disease, stroke, other cerebrovascular disease, diabetes type 1, diabetes type 2, diabetes due to underlying conditions or other specified diabetes, other metabolic disease (excluding diabetes), clinical obesity, clinical underweight, kidney disease, liver disease, blood disorder, dementia, other neurological/musculoskeletal disorder, Down syndrome, and the presence of at least 1 prior molecular or rapid antigen SARS-CoV-2 test record documented in the electronic medical record at least 15 days before the medical encounter date (prevaccination, if vaccinated). VE estimates are not shown for vaccination status comparisons with CIs greater than 50 percentage points around the VE estimate. Adjusted VE could not be calculated for 1 subgroup due to lack of model convergence: hospitalizations, 50-64 years, 2 doses (14-149 days earlier). In vaccination status subgroups with fewer than 10 patients with SARS-CoV-2–positive test results, all numbers in the row were removed because of small cell sizes. ED indicates emergency department; NA, not available; UC, urgent care.

Among all 82 229 included ED and UC encounters, the estimated VE for prior receipt of 2 vaccine doses at least 150 days earlier (median [IQR], 424 [326-470] days) compared with unvaccinated was 28% (95% CI, 24%-31%) for all adults (eFigure 2 in Supplement 1). The estimated VE for a third dose 7 to 119 days earlier was 62% (95% CI, 54%-68%), but the estimated VE for the third dose at least 120 days earlier (median [IQR], 228 [197-257] days) was 32% (95% CI, 29%-36%), similar to that observed with the second dose at least 150 days earlier. Among patients aged 50 years or older eligible for a fourth dose, a fourth dose in the prior 7 to 59 days was associated with higher protection but associated protection also began to decline at 60 days, with a VE closer to null (ie, 0) (Figure 2).

Among hospitalizations, the estimated VE for prior receipt of 2 vaccine doses at least 150 days earlier vs unvaccinated was 25% (95% CI, 17%-32%) (eFigure 2 in Supplement 1). A recent third dose was associated with higher protection (estimated VE, 68% [95% CI, 50%-80%]), but the estimated VE was closer to 0 at 120 days or longer after receipt of vaccine (estimated VE, 36% [95% CI, 29%-42%]), suggestive of waning effectiveness. Among adults aged 65 years or older, a fourth dose was associated with greater protection compared with a late third dose that was similar at 7 to 59 days (estimated VE, 66% [95% CI, 53%-75%]) and 60 days or longer (estimated VE, 57% [95% CI, 44%-66%]) after receipt of the fourth dose (Figure 2). Estimated VEs were similar or higher against COVID-19–related ICU admission or in-hospital death and among patients aged 65 years or older who received a fourth dose (eFigure 3 in Supplement 1). In sensitivity analyses, findings were generally similar and with overlapping CIs between BNT162b2 and mRNA-1273 recipients (eFigures 4-6 in Supplement 1) and when restricted to patients without a documented laboratory-confirmed history of prior SARS-CoV-2 infection (eFigures 7-9 in Supplement 1).

### Comparisons of 3 vs 4 Doses and 2 vs 3 Doses

Receipt of a third dose within the previous 7 to 119 days was associated with greater protection compared with completing 2 doses 150 or more days after receipt among all ED and UC encounters (estimated rVE, 49% [95% CI, 39%-58%]) and hospitalizations (estimated rVE, 57% [95% CI, 35%-72%]) (eTable 4 and eTable 5 in Supplement 1). Likewise, in adults aged 65 years or older, a recent fourth dose was associated with greater protection than a distant third dose among ED and UC encounters (estimated rVE, 35% [95% CI, 28%-41%]) and hospitalizations (estimated rVE, 37% [95% CI, 25%-46%]). There was not enough statistical power to calculate precise estimates for ICU admission or in-hospital death.

### Differences in Characteristics and Outcomes of Hospitalized Patients With COVID-19 by Sublineage Period

In addition to the 3547 hospitalized patients with COVID-19 included during the BA.4 and BA.5 period, there were 12 127 hospitalized patients with COVID-19 during the BA.1 period and 2698 hospitalized patients with COVID-19 during the BA.2 and BA.2.12.1 period (Table 3). Baseline characteristics and outcomes of hospitalized patients with COVID-19 during the BA.4 and BA.5 and BA.2 and BA.2.12.1 periods were similar. However, compared with the BA.1 period, patients hospitalized with COVID-19 during the BA.4 and BA.5 period were older (median [IQR] age, 75 [62-83] vs 67 [54-78] years; SMD, 0.36) and more likely to be vaccinated (2284 patients [64.4%] vs 4349 patients [35.9%]; SMD across vaccination exposure groups, 1.19). The severity of cases during the BA.4 and BA.5 period was lower compared with the BA.1 period, with ICU admission occurring in 459 patients (12.9%) during the BA.4 and BA.5 period vs 2131 patients (17.6%) during the BA.1 period (SMD, 0.13), in-hospital death in 126 patients (3.6%) during the BA.4 and BA.5 period vs 1019 patients (8.4%) during the BA.1 period (SMD, 0.21), and shorter length of stay (median [IQR], 4 [2-7] vs 5 [3-9] days; SMD, 0.31).

**Table 3.** Characteristics of Hospitalized Patients With COVID-19 by Omicron Sublineage Predominant Period

Characteristic	Omicron sublineage predominant period, No. (%)^a^			SMD	
	BA.1	BA.2/BA.2.12.1	BA.4/BA.5	BA.4/BA.5 vs BA.1	BA.4/BA.5 vs BA.2/BA.2.12.1
All hospitalizations	12 127 (100)	2698 (100)	3547 (100)	NA	NA
Site					
Baylor Scott and White Health	3103 (25.6)	359 (13.3)	875 (24.7)	0.24	0.37
Columbia University	569 (4.7)	129 (4.8)	119 (3.4)		
HealthPartners	418 (3.4)	146 (5.4)	151 (4.3)		
Intermountain Healthcare	1063 (8.8)	289 (10.7)	380 (10.7)		
KPNC^b^	2083 (17.2)	846 (31.4)	854 (24.1)		
KPCHR	431 (3.6)	174 (6.4)	103 (2.9)		
PHIX	162 (1.3)	6 (0.2)	14 (0.4)		
Regenstrief Institute	3480 (28.7)	598 (22.2)	892 (25.1)		
University of Colorado	818 (6.7)	151 (5.6)	159 (4.5)		
Age, y					
Median (IQR)	67 (54-78)	75 (63-84)	75 (62-83)	0.36	0.02
18-49	2360 (19.5)	339 (12.6)	431 (12.2)	0.39	0.06
50-64	3062 (25.2)	409 (15.2)	598 (16.9)		
65-74	2698 (22.2)	526 (19.5)	724 (20.4)		
75-84	2430 (20.0)	777 (28.8)	1014 (28.6)		
≥85	1577 (13.0)	647 (24.0)	780 (22.0)		
Sex					
Male	6217 (51.3)	1363 (50.5)	1723 (48.6)	0.05	0.04
Female	5910 (48.7)	1335 (49.5)	1824 (51.4)		
Race and ethnicity					
Hispanic	1872 (15.4)	264 (9.8)	381 (10.7)	0.19	0.12
Non-Hispanic Black	1642 (13.5)	218 (8.1)	374 (10.5)		
Non-Hispanic White	7143 (58.9)	1874 (69.5)	2380 (67.1)		
Non-Hispanic other^c^	901 (7.4)	262 (9.7)	275 (7.8)		
Unknown	569 (4.7)	80 (3.0)	137 (3.9)		
Documented prior SARS-CoV-2 infection					
Yes	648 (5.3)	238 (8.8)	323 (9.1)	0.15	0.01
No	11 479 (94.7)	2460 (91.2)	3224 (90.9)		
mRNA COVID-19 vaccination status					
Unvaccinated	7778 (64.1)	887 (32.9)	1263 (35.6)	1.19	0.37
2 Doses, 14-149 d earlier	351 (2.9)	22 (0.8)	18 (0.5)		
2 Doses, ≥150 d earlier	2943 (24.3)	644 (23.9)	815 (23.0)		
3 Doses, 7-119 d earlier	933 (7.7)	106 (3.9)	33 (0.9)		
3 Doses, ≥120 d earlier	122 (1.0)	905 (33.5)	1099 (31.0)		
4 Doses, 7-59 d earlier	0	110 (4.1)	96 (2.7)		
4 Doses, ≥60 d earlier	0	24 (0.9)	223 (6.3)		
≥1 Chronic respiratory condition^d^					
Yes	8139 (67.1)	1698 (62.9)	2126 (59.9)	0.15	0.06
No	3988 (32.9)	1000 (37.1)	1421 (40.1)		
≥1 Chronic nonrespiratory condition^e^					
Yes	10 328 (85.2)	2427 (90.0)	3129 (88.2)	0.09	0.06
No	1799 (14.8)	271 (10.0)	418 (11.8)		
ICU admission and/or in-hospital death					
Yes	2599 (21.4)	425 (15.8)	523 (14.7)	0.17	0.03
No	9528 (78.6)	2273 (84.2)	3024 (85.3)		
ICU admission					
Yes	2131 (17.6)	364 (13.5)	459 (12.9)	0.13	0.02
No	9996 (82.4)	2334 (86.5)	3088 (87.1)		
Receipt of invasive mechanical ventilation					
Yes	1121 (9.2)	150 (5.6)	215 (6.1)	0.13	0.14
No	8726 (72.0)	2188 (81.1)	2691 (75.9)		
Unknown	2280 (18.8)	360 (13.3)	641 (18.1)		
In-hospital death					
Yes	1019 (8.4)	105 (3.9)	126 (3.6)	0.21	0.02
No	11 108 (91.6)	2593 (96.1)	3421 (96.4)		
Length of hospital stay among survivors, median (IQR), d^f^	5 (3-9)	4 (2-7)	4 (2-7)	0.31	0.12

Abbreviations: ICU, intensive care unit; KPCHR, Kaiser Permanente Center for Health Research; KPNC, Kaiser Permanente Northern California; PHIX, Paso del Norte Health Information Exchange; SMD, standardized mean or proportion difference.

^a^ Sublineage predominance dates are site specific. Dates are reported in eTable 1 in Supplement 1.

^b^ The sample size of hospitalized patients with COVID-19 at KPNC during at least 50% Omicron BA.4/BA.5 sublineage predominance that is reported in this table (854 patients) is different than the sample size reported in Table 1 (890 patients) as a result of multiple hospital admissions occurring within a 30-day period for the same patient (with respect to prior discharge) having been combined separately among hospitalizations already restricted to at least 50% Omicron BA.4/BA.5 sublineage predominance (Table 1) and among all hospitalizations during at least 50% Omicron BA.1, BA.2/BA.2.12.1, and BA.4/BA.5 sublineage predominance periods combined.

^c^ Other race includes Asian, American Indian or Alaska Native, Native Hawaiian or other Pacific Islander, other, and multiple races.

^d^ Chronic respiratory condition was defined as the presence of discharge code for asthma, chronic obstructive pulmonary disease, or other lung disease.

^e^ Chronic nonrespiratory condition was defined as the presence of discharge code for heart failure, ischemic heart disease, hypertension, other heart disease, stroke, other cerebrovascular disease, diabetes type 1 or 2, other diabetes, metabolic disease, clinical obesity, clinically underweight, kidney disease, liver disease, blood disorder, immunosuppression, organ transplant, cancer, dementia, neurologic disorder, musculoskeletal disorder, or Down syndrome.

^f^ Length of hospital stay is reported among patients with no in-hospital death at any point after admission, including among 10 972 hospitalizations during at least 50% Omicron BA.1 sublineage predominance, 2577 during at least 50% Omicron BA.2/BA.2.12.1 sublineage predominance, and 3417 during at least 50% Omicron BA.4/BA.5 sublineage predominance.

## Discussion

In this case-control study using a multistate sample during Omicron BA.4 and BA.5 predominant circulation, first-generation COVID-19 vaccines were associated with effective protection against COVID-19, including for COVID-19–associated hospitalization and ICU admission or in-hospital death. However, protection associated with vaccination declined within several months of the most recent vaccine dose. For hospitalization, estimated VE of 3 doses for all adults and 4 doses for adults aged 50 years or older using an unvaccinated reference group was similar to that reported during BA.2 and BA.2.12.1 predominance.^18^ In addition, changes in the epidemiology of hospitalized patients with COVID-19 were observed; 64% of patients hospitalized with COVID-19 during the BA.4- and BA.5-predominant period had received at least a primary vaccine series, compared with 36% of hospitalized patients during the earlier BA.1-predominant period, aligning with VE findings of lower effectiveness during the BA.4 and BA.5 period. Patients hospitalized during the recent BA.4- and BA.5-predominant period tended to have less severe illness compared with the earlier BA.1 period despite being older. These findings provide an important baseline for bivalent VE analyses.^19,20^

Estimated VE was similar across outcomes, contradicting many past VE studies, including previous studies from the VISION Network, which have tended to show higher vaccine-associated protection for more severe outcomes. This could be due to changes in baseline population immunity (eg, most adults now have evidence of prior infection), changes in behavior (eg, decreased use of social distancing and masks during recent months), or residual confounding.^3,4,21,22,23^ Across all outcomes, estimated VE in this analysis was lower than reported VE when the Delta variant and Omicron BA.1 sublineage predominated.^18,24^ However, the relative contribution of immune evasion from newer variants vs other factors, such as influence of prior infections, on VE is unclear.^25^ A 2022 report from South Africa^26^ found that estimated VE against COVID-19–related hospitalization after receipt of 2 or 3 doses of BNT162b2 waned substantially within several months of vaccine receipt during BA.5-predominant circulation, which is similar to findings in this study. While this analysis did not find waning after the fourth dose, median time from fourth dose to the included encounter in our analysis was less than 3 months (compared with approximately 8 months after the third dose), so VE waning may become evident with increased follow-up time. Approved bivalent booster doses, which include the ancestral strain as well as an additional component targeting BA.4 and BA.5 variants, might provide greater protection against currently circulating variants, although data on VE for bivalent boosters are limited to date, and ongoing surveillance is warranted to guide public health practice and vaccine policy decisions.^19,20^ The implications of these findings on potential vaccine protection for other emerging sublineages, such as XBB sublineages, which carry additional mutations in the spike protein, are unclear.

The finding of less severe disease during BA.4 and BA.5 predominance compared with earlier Omicron sublineage periods has important implications for interpretation of VE over time. Between December 2021 and February 2022, prevalence of infection-induced antibodies among clinical samples tested at commercial laboratories increased from 33.5% to 57.7%, indicating widespread infection-induced immunity by the end of the BA.1 predominant period.^8^ Although VE against infection was substantially lower during Omicron compared with earlier periods, some protection remained, especially individuals who received booster doses, indicating that unvaccinated or unboosted individuals may have higher infection-induced protection compared with individuals who received recommended vaccinations. This increased infection-induced protection in unvaccinated and unboosted individuals, sometimes termed *depletion of susceptibles*, may bias VE estimates, accentuating waning.^27^ VE estimates during BA.4 and BA.5 predominance should therefore be interpreted in the context of population immunity due to prior infection; measured VE is likely blunted by high infection-induced immunity in the unvaccinated and undervaccinated comparison groups, and waning may not be as substantial as estimated.

### Limitations

This analysis has several limitations. First, patient samples were not available for genomic characterization directly. Local prevalence estimates of BA.4 and BA.5, combined with date of testing, were used ecologically to determine inclusion in the analysis periods; however, as estimates of VE during BA.2 and BA.2.12.1 predominance are similar to this analysis, misclassifying some early cases as BA.4 and BA.5 should not have impacted VE estimates substantially. Second, because prior infection was likely underascertained, the primary analysis included all individuals, regardless of documented prior infection or time since documented prior infection, which may have biased results toward the null if prior infection is associated with some protection against reinfection or attenuation of severity if reinfected. Third, although inverse propensity-to-be-vaccinated weights were used to balance vaccinated and unvaccinated medical encounters, residual confounding in VE estimates due to other factors is possible. Fourth, this analysis combined estimated VE against ICU admission and death, which may have obscured differences in VE for these individual outcomes; other severe sequelae of COVID-19, such as postacute sequelae or post–COVID-19 condition were not included.

## Conclusions

This case-control study among immunocompetent adults found that, compared with unvaccinated adults, the estimated VE of recently received third or fourth doses of an mRNA vaccine against ED or UC visits, hospitalization, and ICU admission or death was higher compared with 2 doses but waned during BA.4 and BA.5 variant predominance. Hospitalized patients with COVID-19 were less likely to be admitted to the ICU or experience in-hospital death and had shorter length of stay during BA.4 and BA.5 predominance compared with earlier Omicron sublineage periods.

## Notes

Supplement 1. **eAppendix 1.** Supplementary Methods **eTable 1.** Characteristics of VISION Network Study Sites **eTable 2.** COVID-19–Like Illness Categories and Corresponding *International Classification of Diseases, Ninth Revision* (*ICD-9*), and *International Statistical Classification of Diseases and Related Health Problems, Tenth Revision* (*ICD-10*), Diagnosis Codes **eTable 3.** Covariates with Remaining Imbalances Between Vaccinated and Unvaccinated Patients After Application of Inverse Propensity-to-Be-Vaccinated Weighting for Calculation of Adjusted Odds Ratios **eTable 4.** Relative Vaccine Effectiveness Associated With Protection Against Laboratory-Confirmed COVID-19–Associated Emergency Department or Urgent Care Encounters of 3 vs 2 or 4 vs 3 mRNA COVID-19 Vaccine Doses, by Age Group **eTable 5.** Relative Vaccine Effectiveness Associated With Protection Against Laboratory-Confirmed COVID-19–Associated Hospitalization of 3 vs 2 or 4 vs 3 mRNA COVID-19 Vaccine Doses, by Age Group **eFigure 1.** Flowchart for the Selection of Emergency Department and Urgent Care Encounters **eFigure 2.** Effectiveness Associated With mRNA COVID-19 Vaccine for Protection Against Laboratory-Confirmed COVID-19–Associated Emergency Department or Urgent Care Encounters and Hospitalization Among All Adults **eFigure 3.** Effectiveness Associated With mRNA COVID-19 Vaccine for Protection Against Laboratory-Confirmed COVID-19–Associated Intensive Care Unit Admission and/or In-Hospital Death, by Age Group **eFigure 4.** Effectiveness Associated With mRNA COVID-19 Vaccine for Protection Against Laboratory-Confirmed COVID-19–Associated Emergency Department or Urgent Care Encounters, by mRNA Vaccine Product(s) Received **eFigure 5.** Effectiveness Associated With mRNA COVID-19 Vaccine for Protection Against Laboratory-Confirmed COVID-19–Associated Hospitalization, by mRNA Vaccine Product Received **eFigure 6.** Effectiveness Associated With mRNA COVID-19 Vaccine for Protection Against Laboratory-Confirmed COVID-19–Associated Intensive Care Unit Admission and/or In-Hospital Death, by mRNA Vaccine Product Received **eFigure 7.** Effectiveness Associated With mRNA COVID-19 Vaccine for Protection Against Laboratory-Confirmed COVID-19–Associated Emergency Department or Urgent Care Encounters, by Age Group, Among Patients Without a Prior Documented SARS-CoV-2 Infection **eFigure 8.** Effectiveness Associated With mRNA COVID-19 Vaccine for Protection Against Laboratory-Confirmed COVID-19–Associated Hospitalization, by Age Group, Among Patients Without a Prior Documented SARS-CoV-2 Infection **eFigure 9.** Effectiveness Associated With mRNA COVID-19 Vaccine for Protection Against Laboratory-Confirmed COVID-19–Associated Intensive Care Unit Admission and/or In-Hospital Death, by Age Group, Among Patients Without a Prior Documented SARS-CoV-2 Infection

Supplement 2.
Data Sharing Statement
