# Supplementary material for: Estimation of COVID-19 mRNA Vaccine Effectiveness and COVID-19 Illness and Severity by Vaccination Status During Omicron BA.4 and BA.5 Sublineage Periods
Source: JAMA Netw Open. 2023 Mar 15;6(3):e232598. doi: 10.1001/jamanetworkopen.2023.2598 (PMC10018321; doi:10.1001/jamanetworkopen.2023.2598)
Supplement: Supplement 1. — eAppendix 1. Supplementary Methods eTable 1. Characteristics of VISION Network Study Sites eTable 2. COVID-19–Like Illness Categories and Corresponding International Classification of Diseases, Ninth Revision (ICD-9), and International Statistical Classification of Diseases and Related Health Problems, Tenth Revision (ICD-10), Diagnosis Codes eTable 3. Covariates with Remaining Imbalances Between Vaccinated and Unvaccinated Patients After Application of Inverse Propensity-to-Be-Vaccinated Weighting for Calculation of Adjusted Odds Ratios eTable 4. Relative Vaccine Effectiveness Associated With Protection Against Laboratory-Confirmed COVID-19–Associated Emergency Department or Urgent Care Encounters of 3 vs 2 or 4 vs 3 mRNA COVID-19 Vaccine Doses, by Age Group eTable 5. Relative Vaccine Effectiveness Associated With Protection Against Laboratory-Confirmed COVID-19–Associated Hospitalization of 3 vs 2 or 4 vs 3 mRNA COVID-19 Vaccine Doses, by Age Group eFigure 1. Flowchart for the Selection of Emergency Department and Urgent Care Encounters eFigure 2. Effectiveness Associated With mRNA COVID-19 Vaccine for Protection Against Laboratory-Confirmed COVID-19–Associated Emergency Department or Urgent Care Encounters and Hospitalization Among All Adults eFigure 3. Effectiveness Associated With mRNA COVID-19 Vaccine for Protection Against Laboratory-Confirmed COVID-19–Associated Intensive Care Unit Admission and/or In-Hospital Death, by Age Group eFigure 4. Effectiveness Associated With mRNA COVID-19 Vaccine for Protection Against Laboratory-Confirmed COVID-19–Associated Emergency Department or Urgent Care Encounters, by mRNA Vaccine Product(s) Received eFigure 5. Effectiveness Associated With mRNA COVID-19 Vaccine for Protection Against Laboratory-Confirmed COVID-19–Associated Hospitalization, by mRNA Vaccine Product Received eFigure 6. Effectiveness Associated With mRNA COVID-19 Vaccine for Protection Against Laboratory-Confirmed COVID-19–Associated Intensive Care Unit Adm [file jamanetwopen-e232598-s001.pdf]

Supplemental Online Content

Link-Gelles R, Levy ME, Natarajan K, et al. Estimation of COVID-19 mRNA vaccine effectiveness and COVID-19 illness and severity by vaccination status during Omicron BA.4 and BA.5 sublineage periods. *JAMA Netw Open*. 2023;6(3):e232598. doi:10.1001/jamanetworkopen.2023.2598

eAppendix 1. Supplementary Methods
eTable 1. Characteristics of VISION Network Study Sites
eTable 2. COVID-19–Like Illness Categories and Corresponding *International Classification of Diseases, Ninth Revision* (*ICD-9*), and *International Statistical Classification of Diseases and Related Health Problems, Tenth Revision* (*ICD-10*), Diagnosis Codes
eTable 3. Covariates with Remaining Imbalances Between Vaccinated and Unvaccinated Patients After Application of Inverse Propensity-to-Be-Vaccinated Weighting for Calculation of Adjusted Odds Ratios
eTable 4. Relative Vaccine Effectiveness Associated With Protection Against Laboratory-Confirmed COVID-19–Associated Emergency Department or Urgent Care Encounters of 3 vs 2 or 4 vs 3 mRNA COVID-19 Vaccine Doses, by Age Group
eTable 5. Relative Vaccine Effectiveness Associated With Protection Against Laboratory-Confirmed COVID-19–Associated Hospitalization of 3 vs 2 or 4 vs 3 mRNA COVID-19 Vaccine Doses, by Age Group
eFigure 1. Flowchart for the Selection of Emergency Department and Urgent Care Encounters
eFigure 2. Effectiveness Associated With mRNA COVID-19 Vaccine for Protection Against Laboratory-Confirmed COVID-19–Associated Emergency Department or Urgent Care Encounters and Hospitalization Among All Adults
eFigure 3. Effectiveness Associated With mRNA COVID-19 Vaccine for Protection Against Laboratory-Confirmed COVID-19–Associated Intensive Care Unit Admission and/or In-Hospital Death, by Age Group
eFigure 4. Effectiveness Associated With mRNA COVID-19 Vaccine for Protection Against Laboratory-Confirmed COVID-19–Associated Emergency Department or Urgent Care Encounters, by mRNA Vaccine Product(s) Received
eFigure 5. Effectiveness Associated With mRNA COVID-19 Vaccine for Protection Against Laboratory-Confirmed COVID-19–Associated Hospitalization, by mRNA Vaccine Product Received
eFigure 6. Effectiveness Associated With mRNA COVID-19 Vaccine for Protection Against Laboratory-Confirmed COVID-19–Associated Intensive Care Unit Admission and/or In-Hospital Death, by mRNA Vaccine Product Received
eFigure 7. Effectiveness Associated With mRNA COVID-19 Vaccine for Protection Against Laboratory-Confirmed COVID-19–Associated Emergency Department or Urgent Care Encounters, by Age Group, Among Patients Without a Prior Documented SARS-CoV-2 Infection
eFigure 8. Effectiveness Associated With mRNA COVID-19 Vaccine for Protection Against Laboratory-Confirmed COVID-19–Associated Hospitalization, by Age Group, Among Patients Without a Prior Documented SARS-CoV-2 Infection
eFigure 9. Effectiveness Associated With mRNA COVID-19 Vaccine for Protection Against Laboratory-Confirmed COVID-19–Associated Intensive Care Unit Admission and/or In-Hospital Death, by Age Group, Among Patients Without a Prior Documented SARS-CoV-2 Infection

This supplemental material has been provided by the authors to give readers additional information about their work.

© 2023 Link-Gelles R et al. *JAMA Network Open.*

eAppendix. Supplementary Methods

Section 1: Extracting Percent Positivity Data from HHS Protect

National laboratory testing data, including data on the state and county level, are available on the password-protected HHS Protect Public Data Hub. The laboratory testing data include viral SARS-CoV-2 laboratory test results (reverse transcription polymerase chain reaction [RT-PCR]) from over 1,000 United States laboratories and testing locations including commercial and reference laboratories, public health laboratories, hospital laboratories, and other testing locations. Data presented in HHS Protect are representative of diagnostic specimens being tested and reflect the majority of, but not all, SARS-CoV-2 laboratory-based testing conducted in the United States. Data from HHS Protect are electronic health records and do not contain personally identifiable information (see https://www.hhs.gov/sites/default/files/hhs-protect-faqs.pdf for more information).

For this analysis, daily laboratory testing data were downloaded from HHS Protect and aggregated at the county-level by date of report. Using the county-level average of the daily percentage of tests that were positive during each day and the prior six days, we further aggregated and computed each date's daily seven-day average separately for each site geographic sub-region (aggregates of counties) by taking into account county population sizes. This population-weighted daily value for each site sub-region was then assigned to each medical encounter as a measure of local SARS-CoV-2 circulation based on the medical encounter index date and site sub-region of the medical facility within which the respective encounter occurred.

Section 2: Statistical Methods

Section 2.1: Overview

This section further expands on the statistical methodology that was used to estimate the association of symptomatic laboratory-confirmed SARS-CoV-2 infection in an emergency department (ED) or urgent care (UC) clinic setting or hospital setting with vaccination status. In the context of a test-negative case-control study conducted during a period of ≥50% Omicron BA.4/BA.5 sublineage predominance among medical encounters for patients with COVID-19–like illness, the odds of having each specific vaccination status (each defined based on the number of doses received and number of days since the most recent dose) versus unvaccinated status was compared between SARS-CoV-2–positive cases and SARS-CoV-2–negative controls. The test-negative design can minimize biases associated with access to vaccines and healthcare seeking behaviors and has been used extensively to estimate vaccine effectiveness (VE) against medically attended influenza virus illness (1,2). Methods for the current analysis were based on those of prior VISION Network analyses, which have been detailed elsewhere (3). Analyses were conducted separately among ED or UC encounters, hospitalizations, and hospitalizations with intensive care unit (ICU) admission and/or in-hospital death ≤28 days after admission. Analyses were also conducted separately for each pairwise vaccination status comparison (e.g., 3 doses with 3rd dose 7-119 days earlier versus unvaccinated). Both inverse propensity score weighting and covariate adjustment procedures were used to control for confounding, that is, control for differences in characteristics between vaccinated and unvaccinated patients when estimating odds ratios for each pairwise comparison, which were then used to estimate VE in each setting using the formula VE = [1−adjusted OR] x 100%.

Section 2.2: Repeat Encounters

Although data were collected and analyzed at the encounter level rather than at the individual patient level, repeat encounters among unique patients were not expected to be highly prevalent given the relatively short time period examined. In general, when the percentage of repeat encounters (for patients who previously contributed an encounter) exceeds 10%, a sensitivity analysis is conducted to assess the effect of within-person correlation on ORs and confidence intervals. In this analysis of ED/UC encounters and hospitalizations during a period of ≥50% Omicron BA.4/BA.5 sublineage predominance, there were 8.5% (n=6,364) repeat ED/UC encounters and 0.4% (n=91) repeat hospitalizations among patients with a prior encounter of the respective type; thus, no further action was taken.

Section 2.3: Inverse Propensity Score Weighting

© 2023 Link-Gelles R et al. *JAMA Network Open.*

Using established methods for estimating propensity scores within case-control studies (4), we first estimated propensity-for-vaccination scores among SARS-CoV–negative controls, with potential confounding variables used as independent variables and vaccination status as the dependent variable. Next, the fitted model was used to calculate propensity-for-vaccination scores for SARS-CoV-2–positive cases. Because each vaccinated category was compared with unvaccinated status in separate analyses, the propensity score represented the estimated probability of being in the specific vaccination category of interest versus being unvaccinated, conditional on measured covariates representing potential confounding variables. Finally, in primary multivariable regression models to estimate the association between symptomatic medically attended laboratory-confirmed SARS-CoV-2 infection and vaccination status, vaccinated patients were weighted by the inverse of their propensity to be vaccinated and unvaccinated patients were weighted by the inverse of their propensity to not be vaccinated. Inverse propensity score weighting was designed to estimate an overall average treatment effect.

Propensity to be vaccinated was estimated using boosted regression trees (BRT), a nonparametric sequential regression technique (4). Regularization settings to prevent overfitting by BRT methods were determined based on overall sample size; however, the following guidelines were followed: shallow tree depth (2-3 interaction levels), large number of trees (7,000), low learning rate (0.01), and 75% bagging.

Among a set of measured covariates that were identified as potential confounders, those included in the propensity score model were covariates empirically determined to be associated with both the outcome (case-control status) and exposure (vaccination status), with significant differences between groups defined as those with an absolute standardized mean or proportion difference >0.10. The following socio-demographic, facility, and medical factors were considered for inclusion: age, sex, race, ethnicity, Medicaid status, calendar date (number of days since January 1, 2021 based on medical encounter index date), geographic region (based on sub-regions defined for each site), local SARS-CoV-2 circulation on the day of each medical encounter index date, urban-rural classification of facility, hospital type (hospital setting only), number of hospital beds (hospital setting only), chronic respiratory condition, chronic non-respiratory condition, asthma, chronic obstructive pulmonary disease, other chronic lung disease, heart failure, ischemic heart disease, hypertension, other heart disease, stroke, other cerebrovascular disease, diabetes type 1, diabetes type 2, diabetes due to underlying conditions or other specified diabetes, other metabolic disease (excluding diabetes), clinical obesity, clinical underweight, renal disease, liver disease, blood disorder, dementia, other neurological/musculoskeletal disorder, Down syndrome, and the presence of at least one prior molecular or rapid antigen SARS-CoV-2 test record documented in the electronic medical record ≥15 days before the medical encounter index date (pre-vaccination, if vaccinated). Four covariates were included in the propensity score model regardless of their association with the outcome and exposure: age, calendar date, geographic region, and local SARS-CoV-2 circulation on the day of each medical encounter index date.

Applying best practices for inverse probability of treatment weights described by Austin and Stuart (5), the distributions of weights were examined for each vaccination status comparison in each medical setting. In each subgroup, outlying weights were identified at the extreme upper end of the distribution. Therefore, we truncated weights at the 99th percentile for each subgroup. Propensity scores and weights were calculated using the 'twang' R package (6). All propensity score analyses were conducted using R version 4.1.2.

Section 2.4: Primary Outcome Model and Covariate Adjustment

The primary outcome model to estimate the association between symptomatic medically attended laboratory-confirmed SARS-CoV-2 infection and vaccination status was a multivariable logistic regression model, with SARS-CoV-2 test result (i.e., case-control status) as the dependent variable and vaccination status as a dichotomous independent variable. Medical encounter observations were weighted by their inverse propensity to be vaccinated (if vaccinated) or unvaccinated (if not vaccinated). Four covariates were also directly included as additional independent variables in the regression model to account for possible residual confounding that remained after inverse propensity score weighting based on BRT modeling. The four variables were age (as a spline), calendar date (as spline), geographic region, and local SARS-CoV-2 circulation on the day of each medical encounter index date (as a spline). Spline functions for calendar date, local SARS-CoV-2 circulation, and age were defined as natural cubic splines with knots at quartiles. In addition, any other covariates with distributions that remained imbalanced between vaccinated and unvaccinated patients after inverse propensity score weighting, based on an absolute

© 2023 Link-Gelles R et al. *JAMA Network Open.*

standardized mean or proportion difference >0.2, were also included directly in the respective regression model. The list of unbalanced variables for each model is presented in eTable 3.

Section 2.5: Relative VE

In addition to calculating ORs to estimate absolute VE (i.e., VE for receipt of vaccine compared with unvaccinated status), ORs were also calculated to estimate relative VE, for which a specific vaccinated group was compared with a different vaccinated group in order to determine the incremental benefit of receiving an additional vaccine dose when recommend. Relative VE was estimated by comparing individuals who had recently received a 3rd or 4th dose to those who were eligible for, but had not received, the 3rd or 4th dose, respectively. More specifically, the two comparisons were: (1) 3 doses with the 3rd dose in the last 7-119 days versus 2 doses with the 2nd dose ≥150 days earlier; and, among patients aged ≥50 years, (2) 4 doses with the 4th dose within the last 7-119 days versus 3 doses with the 3rd dose ≥120 days earlier.

To calculate ORs reflecting relative VE comparisons, with receipt of 2 or 3 doses serving as the referent group, a similar methodology was used: patient encounters were weighted based on their inverse propensity to be 3-dose vaccinated (if 3-dose vaccinated) or 2-dose vaccinated (if 2-dose vaccinated) – or 4-dose vaccinated (if 4-dose vaccinated) or 3-dose vaccinated (if 3-dose vaccinated) – and a dichotomous variable for vaccination status (3- versus 2-dose vaccinated or 4- versus 3-dose vaccinated) was included as the independent variable used in primary outcome models.

Section 2.6: Subgroup Analyses

The analyses described were conducted in each setting (ED or UC encounters, hospitalizations, and hospitalizations with ICU admission and/or in-hospital death) and in different subgroups within each setting. Analyses were conducted among all adults aged ≥18 years as well as separately among three age groups (18-49, 50-64, and ≥65 years). They were also conducted separately for each vaccine product(s) received (mRNA-1273 [Moderna], BNT162b2 [Pfizer-BioNTech], and heterologous pattern) and among patients without prior infection documented in the electronic medical record ≥15 days prior to the hospital admission or encounter date. Propensity score weights and OR estimates for each vaccination status comparison were only calculated using patient encounters qualifying for inclusion in the respective subgroup.

References

1. Foppa IM, Haber M, Ferdinands JM, Shay DK. The case test-negative design for studies of the effectiveness of influenza vaccine. Vaccine. 2013; 31:3104–9.
2. Jackson ML, Nelson JC. The test-negative design for estimating influenza vaccine effectiveness. Vaccine.2013; 31:2165–8.
3. Thompson MG, Stenehjem E, Grannis S, Ball S, et al. Effectiveness of COVID-19 vaccines in ambulatory and inpatient care settings. 2011; 385(15): 1355-71.
4. McCaffrey DF, Ridgeway G, Morral AR. Propensity score estimation with boosted regression for evaluating causal effects in observational studies. Psychological methods. 2004; 9(4): 403.
5. Austin PC, Stuart, EA. Moving towards best practices when using inverse probability of treatment weights (IPTW) using the propensity score to estimate causal treatment effects in observational studies. Statistics and Medicine, 2015; 34: 3661-3679.
6. Ridgeway G, McCaffrey D, Morral AR, Burgette L, Grriffin BA. Toolkit for Weighting and Analysis of Nonequivalent Groups: A tutorial for the twang package. Santa Monica, CA: RAND Corporation; 2017.

© 2023 Link-Gelles R et al. *JAMA Network Open.*

eTable 1. Characteristics of VISION Network Study Sites

Network partner (state)	Geographic sub-regions[a] (N=45), No.	Hospitals (N=268), No.	EDs (N=292), No.	Urgent care clinics (N=140), No.	≥50% Omicron BA.1 sublineage predominance period	≥50% Omicron BA.2/ BA.2.12.1 sublineage predominance period	≥50% Omicron BA.4/BA.5 sublineage predominance period	Source of vaccination records	Vaccine record lag[b]
Baylor Scott & White Health (Texas)	9[c]	26	29	7	12/16/21–3/18/22	3/19–6/21/22	6/22–8/20/22	ImmTrac Texas Immunization Registry and EHRs	3 days
Columbia University Irving Medical Center (New York)	1	3	2	N/A	12/18/21–3/16/22	3/17–6/28/22	6/29–8/20/22	New York Citywide Immunization Registry and EHRs	1 week
HealthPartners (Minnesota and Wisconsin)	2	10	10	23	12/25/21–3/21/22	3/22–6/21/22	6/22–8/20/22	Minnesota Immunization Information Connection and EHRs	1 week
Intermountain Healthcare (Utah)	8	22	22	33	12/24/21–3/18/22	3/19–6/22/22	6/23–8/20/22	Utah State Immunization Information System and EHRs	1 week
Kaiser Permanente Northern California (California)	8	29	29	N/A	12/21/21–3/20/22	3/21–6/24/22	6/25–8/20/22	California Immunization Registry, CARE Everywhere[d], pharmacy data, claims data, and EHRs	2 weeks

© 2023 Link-Gelles R et al. *JAMA Network Open.*

Network partner (state)	Geographic sub-regions[a] (N=45), No.	Hospitals (N=268), No.	EDs (N=292), No.	Urgent care clinics (N=140), No.	≥50% Omicron BA.1 sublineage predominance period	≥50% Omicron BA.2/ BA.2.12.1 sublineage predominance period	≥50% Omicron BA.4/BA.5 sublineage predominance period	Source of vaccination records	Vaccine record lag[b]
Kaiser Permanente Northwest (Oregon and Washington)	3	57	97	57	12/24/21–3/23/22	3/24–6/28/22	6/29–8/20/22	Oregon Immunization Information System, Washington State Immunization Information System, claims data, and EHRs	2 weeks
Paso del Norte Health Information Exchange (Texas)	1	7	N/A	N/A	12/29/21–3/29/22	3/30–6/21/22	6/22–8/20/22	EHRs	1 week
Regenstrief Institute (Indiana)	10[e]	102	103	N/A	12/26/21–3/20/22	3/21–6/18/22	6/19–8/20/22	Children and Hoosier Immunization Registry Program	1 week
University of Colorado (Colorado)	3	12	N/A	20	12/19/21–3/20/22	3/21–6/18/22	6/19–8/20/22	Colorado Immunization Information System and EHRs	1 week

Abbreviations: ED, emergency department; EHRs, electronic health records; N/A, not applicable; UC, urgent care.

[a] Each site defined sub-regions that represent meaningfully distinct geographic areas within their network. Sub-region values were assigned to medical encounters based on the location of the admitting hospital, ED, or UC clinic and were used for purposes of adjustment for geographic region in multivariable regression modeling.

[b] The vaccine record lag is the duration of time post-vaccination before vaccine records are expected to be available in sites' records contributing to data collection for this study.

[c] For Baylor Scott & White Health, inpatient facilities were located in only 8 of the 9 sub-regions.

[d] CARE Everywhere is an Epic electronic health record inter-hospital system for vaccination record sharing.

[e] For Regenstrief Institute, one of the 10 sub-region values represents unknown location of facility, and inpatient facilities were located in only 8 of the 9 defined sub-regions.

© 2023 Link-Gelles R et al. *JAMA Network Open.*

eTable 2. COVID-19–Like Illness Categories and Corresponding *International Classification of Diseases, Ninth Revision* (*ICD-9*), and *International Statistical Classification of Diseases and Related Health Problems, Tenth Revision* (*ICD-10*), Diagnosis Codes

**Description of Diagnosis**	**ICD-10 codes**	**ICD-9 codes**
**COVID-19 Pneumonia**		
Pneumonia due to SARS-associated coronavirus	J12.81	N/A
Pneumonia due to coronavirus disease 2019	J12.82	N/A
**Influenza Pneumonia**		
Influenza due to identified novel influenza A virus with pneumonia	J09.X1	488.81
Influenza due to other identified influenza virus with pneumonia	J10.0*	N/A
Influenza due to other identified influenza virus with unspecified type of pneumonia	J10.00	487.0
Influenza due to other identified influenza virus with the same other identified influenza virus pneumonia	J10.01	487.0
Influenza due to other identified influenza virus with other specified pneumonia	J10.08	487.0, 488.11
Influenza due to unidentified influenza virus with pneumonia	J11.0*	N/A
Influenza due to unidentified influenza virus with unspecified type of pneumonia	J11.00	487.0
Influenza due to unidentified influenza virus with specified pneumonia	J11.08	487.0
Influenza with pneumonia	N/A	487*
**Other Viral Pneumonia**	J12.0, J12.1, J12.3, J12.3, J12.89, J12.9	480*
**Bacterial and Other Pneumonia**		
Streptococcus pneumoniae pneumonia	J13	481
Hemophilus influenzae pneumonia	J14	482.2
Other bacterial pneumonia	J15*	482*
Pneumonia due to other specified organism	J16*	483*
Pneumonia in infectious diseases classified elsewhere	J17	484*
Pneumonia, unspecified organism	J18*	486
**Influenza Disease**	J09.X2, J09.X3, J09.X9, J10.1, J10.2, J10.8*, J11.1, J11.2, J11.8*	488*
**Acute respiratory distress syndrome**	J80	518.82
**COPD with acute exacerbation**	J44.1	491.21
**Asthma acute exacerbation**	J45.21, J45.22, J45.31, J45.32, J45.41, J45.42, J45.51, J45.52, J45.901, J45.902	493.01, 493.02, 493.11, 493.12, 493.21, 493.22, 493.91, 493.92

© 2023 Link-Gelles R et al. *JAMA Network Open.*

Description of Diagnosis	ICD-10 codes	ICD-9 codes
**Respiratory failure**		
Acute respiratory failure	J96.0*	518.81
Acute and chronic respiratory failure	J96.2*	518.84
Respiratory arrest	R09.2	799.1
**Other acute lower respiratory tract infections**		
Acute bronchitis	J20*	466.0
Acute bronchiolitis	J21*	466.1*
Unspecified acute lower respiratory infection	J22	519.8
Bronchitis, not specified as acute or chronic	J40	490
COPD with acute lower respiratory infection	J44.0	491.22
Simple and mucopurulent chronic bronchitis	J41*	491*
Unspecified chronic bronchitis	J42	491.9
Emphysema	J43*	492*
Bronchiectasis	J47*	494*
Abscess of lung and mediastinum	J85*	513*
Gangrene and necrosis of lung	J85.0	N/A
Abscess of lung without pneumonia	J85.2	513.0
Abscess of mediastinum	J85.3	513.1
Abscess of lung with pneumonia	J85.1	513.0
Pyothorax	J86*	510*
**Acute and chronic sinusitis**	J01* , J32*	461* , 473*
**Acute upper respiratory tract infections**	J00*, J02*, J03*, J04*, J05*, J06*	460*, 462, 463, 464*, 465*
**Signs and symptoms of acute respiratory illness**		
Hemoptysis	R04.2	786.3
Cough	R05 R05.1, R05.2, R05.4, R05.8, R05.9	786.2
Dyspnea unspecified	R06.00	786.09
Shortness of breath	R06.02	786.05
Acute respiratory distress	R06.03	N/A
Stridor	R06.1	786.1
Wheezing	R06.2	786.07
Other abnormalities of breathing	R06.8	N/A
Apnea, not elsewhere classified	R06.81	786.03
Tachypnea, not elsewhere classified	R06.82	786.06
Other abnormalities of breathing/ Other symptoms involving head & neck	R06.89	784.99
Other dyspnea and respiratory abnormality	N/A	786.09
Other symptoms involving respiratory system and chest	N/A	786.9

© 2023 Link-Gelles R et al. *JAMA Network Open.*

Description of Diagnosis	ICD-10 codes	ICD-9 codes
Chest pain on breathing/ painful respiration	R07.1	786.52
Asphyxia and hypoxemia	R09.0*	N/A
Asphyxia	R09.01	799.01
Hypoxemia	R09.02	799.02
Pleurisy	R09.1	511.0
Respiratory arrest	R09.2	799.1
Abnormal sputum	R09.3	786.4
Other specified symptoms and signs involving the circulatory and respiratory systems	R09.8*	478.19, 784.91, 786.7
**Signs and symptoms of acute febrile illness**		
Fever	R50*	N/A
Fever presenting with conditions classified elsewhere	R50.81	780.61
Fever unspecified	R50.9	780.6
Chills (without fever)	R68.83	780.64
**Signs and symptoms of acute non-respiratory illness**		
Diarrhea	R19.7	787.91
Disturbance of smell and taste	R43*	N/A
Unspecified disturbances of smell and taste	R43.9	781.1, V41.5
Headache	R51.9	784.0
Myalgia	M79.10, M79.18	729.1
Sepsis - Symptoms and signs specifically associated with systemic inflammation and infection	R65*	785.52
Other malaise	R53.81	780.79
Other fatigue	R53.83	780.79
Shock, unspecified	R57.9	785.5
Debility unspecified	N/A	799.3
Altered level of consciousness / altered mental status	R41.82, R40.0, R40.1	780.97, 780.0*
Weakness	R53.1	780.79
Nausea and Vomiting	R11.0, R11.10, R11.11, R11.15, R11.2	787*
Rash and other nonspecific skin eruption	R21*	782.1
Abdominal pain	R10.0, R10.1*, R10.2, R10.3*, R10.81*, R10.84, R10.9	789*

Abbreviations: COPD, Chronic Obstructive Pulmonary Disease; ICD-10, *International Classification of Diseases, 10th Revision*; ICD-9, *International Classification of Diseases, 9th Revision*; N/A, not applicable.
*Includes all sub-codes.

eTable 3. Covariates with Remaining Imbalances Between Vaccinated and Unvaccinated Patients After Application of Inverse Propensity-to-Be-Vaccinated Weighting for Calculation of Adjusted Odds Ratios

**Vaccination status (each compared with unvaccinated)**	**Setting**	**Age group, y**	**Covariates with absolute SMD >0.2 after weighting[a]**
2 doses (14-149 days earlier)	ED or UC encounters	≥18	None
2 doses (≥150 days earlier)	ED or UC encounters	≥18	None
3 doses (7-119 days earlier)	ED or UC encounters	≥18	None
3 doses (≥120 days earlier)	ED or UC encounters	≥18	None
2 doses (14-149 days earlier)	ED or UC encounters	18-49	None
2 doses (≥150 days earlier)	ED or UC encounters	18-49	None
3 doses (7-119 days earlier)	ED or UC encounters	18-49	None
3 doses (≥120 days earlier)	ED or UC encounters	18-49	None
2 doses (14-149 days earlier)	ED or UC encounters	50-64	None
2 doses (≥150 days earlier)	ED or UC encounters	50-64	None
3 doses (7-119 days earlier)	ED or UC encounters	50-64	None
3 doses (≥120 days earlier)	ED or UC encounters	50-64	None
4 doses (7-59 days earlier)	ED or UC encounters	50-64	Hypertension (SMD=0.21)
4 doses (≥60 days earlier)	ED or UC encounters	50-64	Presence of prior SARS-CoV-2 test record (SMD=0.25)
2 doses (14-149 days earlier)	ED or UC encounters	≥65	None
2 doses (≥150 days earlier)	ED or UC encounters	≥65	None
3 doses (7-119 days earlier)	ED or UC encounters	≥65	None
3 doses (≥120 days earlier)	ED or UC encounters	≥65	None
4 doses (7-59 days earlier)	ED or UC encounters	≥65	None
4 doses (≥60 days earlier)	ED or UC encounters	≥65	None
2 doses (14-149 days earlier)	Hospitalizations	≥18	None
2 doses (≥150 days earlier)	Hospitalizations	≥18	None
3 doses (7-119 days earlier)	Hospitalizations	≥18	None
3 doses (≥120 days earlier)	Hospitalizations	≥18	None
2 doses (14-149 days earlier)	Hospitalizations	18-49	Medicaid status (SMD=0.25)
2 doses (≥150 days earlier)	Hospitalizations	18-49	None
3 doses (7-119 days earlier)	Hospitalizations	18-49	Chronic non-respiratory condition (SMD=0.40), hypertension (SMD=0.24), other metabolic disease (SMD=0.24)
3 doses (≥120 days earlier)	Hospitalizations	18-49	None
2 doses (14-149 days earlier)	Hospitalizations	50-64	Medicaid status (SMD=0.24)
2 doses (≥150 days earlier)	Hospitalizations	50-64	None
3 doses (7-119 days earlier)	Hospitalizations	50-64	None
3 doses (≥120 days earlier)	Hospitalizations	50-64	None

© 2023 Link-Gelles R et al. *JAMA Network Open.*

Vaccination status (each compared with unvaccinated)	Setting	Age group, y	Covariates with absolute SMD >0.2 after weighting[a]
4 doses (7-59 days earlier)	Hospitalizations	50-64	Medicaid status (SMD=0.34), heart failure (SMD=0.28)
4 doses (≥60 days earlier)	Hospitalizations	50-64	Race (SMD=0.29), Medicaid status (SMD=0.24), other chronic lung disease (SMD=0.27), heart failure (SMD=0.26)
2 doses (14-149 days earlier)	Hospitalizations	≥65	Chronic obstructive pulmonary disease (SMD=0.33)
2 doses (≥150 days earlier)	Hospitalizations	≥65	None
3 doses (7-119 days earlier)	Hospitalizations	≥65	Presence of prior SARS-CoV-2 test record (SMD=0.30)
3 doses (≥120 days earlier)	Hospitalizations	≥65	None
4 doses (7-59 days earlier)	Hospitalizations	≥65	None
4 doses (≥60 days earlier)	Hospitalizations	≥65	None
2 doses (14-149 days earlier)	ICU admission or in-hospital death	≥18	None
2 doses (≥150 days earlier)	ICU admission or in-hospital death	≥18	None
3 doses (7-119 days earlier)	ICU admission or in-hospital death	≥18	None
3 doses (≥120 days earlier)	ICU admission or in-hospital death	≥18	None
2 doses (14-149 days earlier)	ICU admission or in-hospital death	18-49	Medicaid status (SMD=0.25)
2 doses (≥150 days earlier)	ICU admission or in-hospital death	18-49	None
3 doses (7-119 days earlier)	ICU admission or in-hospital death	18-49	Medicaid status (SMD=0.20), chronic non-respiratory condition (SMD=0.35), hypertension (SMD=0.24), other heart disease (SMD=0.26)
3 doses (≥120 days earlier)	ICU admission or in-hospital death	18-49	None
2 doses (14-149 days earlier)	ICU admission or in-hospital death	50-64	Chronic respiratory condition (SMD=0.21), presence of prior SARS-CoV-2 test record (SMD=0.35)
2 doses (≥150 days earlier)	ICU admission or in-hospital death	50-64	None
3 doses (7-119 days earlier)	ICU admission or in-hospital death	50-64	Sex (SMD=0.21), hospital type (SMD=0.34)
3 doses (≥120 days earlier)	ICU admission or in-hospital death	50-64	None
4 doses (7-59 days earlier)	ICU admission or in-hospital death	50-64	Medicaid status (SMD=0.36), heart failure (SMD=0.29), ischemic heart disease (SMD=0.21), presence of prior SARS-CoV-2 test record (SMD=0.37)
4 doses (≥60 days earlier)	ICU admission or in-hospital death	50-64	Race (SMD=0.29), Medicaid status (SMD=0.24), other chronic lung disease (SMD=0.28), heart failure (SMD=0.26), clinical underweight (SMD=0.21), other neurological/musculoskeletal disorder (SMD=0.34), presence of prior SARS-CoV-2 test record (SMD=0.36)
2 doses (14-149 days earlier)	ICU admission or in-hospital death	≥65	Sex (SMD=0.26), chronic obstructive pulmonary disease (SMD=0.21), other neurological/musculoskeletal disorder (SMD=0.36)
2 doses (≥150 days earlier)	ICU admission or in-hospital death	≥65	None

© 2023 Link-Gelles R et al. *JAMA Network Open.*

Vaccination status (each compared with unvaccinated)	Setting	Age group, y	Covariates with absolute SMD >0.2 after weighting[a]
3 doses (7-119 days earlier)	ICU admission or in-hospital death	≥65	Presence of prior SARS-CoV-2 test record (SMD=0.31)
3 doses (≥120 days earlier)	ICU admission or in-hospital death	≥65	None
4 doses (7-59 days earlier)	ICU admission or in-hospital death	≥65	None
4 doses (≥60 days earlier)	ICU admission or in-hospital death	≥65	None

Abbreviations: ED, emergency department; ICU, intensive care unit; SMD, standardized mean or proportion difference; UC, urgent care.

[a] Covariates included as independent variables in all primary outcome multivariable regression models were age (as a spline), calendar date (as spline), geographic region, and local SARS-CoV-2 circulation on the day of each medical encounter index date (as a spline). Additional covariates evaluated for imbalances after inverse propensity-to-be-vaccinated weighting included sex, race, ethnicity, Medicaid status, urban-rural classification of facility, hospital type (if relevant), number of hospital beds (if relevant), chronic respiratory condition, chronic non-respiratory condition, asthma, chronic obstructive pulmonary disease, other chronic lung disease, heart failure, ischemic heart disease, hypertension, other heart disease, stroke, other cerebrovascular disease, diabetes type 1, diabetes type 2, diabetes due to underlying conditions or other specified diabetes, other metabolic disease (excluding diabetes), clinical obesity, clinical underweight, renal disease, liver disease, blood disorder, dementia, other neurological/musculoskeletal disorder, Down syndrome, and the presence of at least one prior molecular or rapid antigen SARS-CoV-2 test record documented in the electronic medical record ≥15 days before the medical encounter date (pre-vaccination, if vaccinated). An absolute SMD >0.20 indicated a non-negligible difference in variable distributions between vaccinated and unvaccinated patients. All covariates with an absolute SMD >0.20 after weighting were also included in primary regression models for the respective odds ratio and vaccine effectiveness estimate(s) to minimize residual confounding.

© 2023 Link-Gelles R et al. *JAMA Network Open.*

eTable 4. Relative Vaccine Effectiveness Associated With Protection Against Laboratory-Confirmed COVID-19–Associated Emergency Department or Urgent Care Encounters of 3 vs 2 or 4 vs 3 mRNA COVID-19 Vaccine Doses, by Age Group

**Encounter type/comparison type/age group/specific vaccination status comparison**	**Total**	**SARS-CoV-2–positive, No. (%)**	**Days since recent dose, median (IQR)**	**Adjusted relative VE[a] % (95% CI)**
**ED or UC encounters**				
**Comparisons to assess incremental benefit of additional dose when recommended**				
**All adults**				
**3 doses vs. 2 doses**				
2 doses ≥150 days earlier (Ref)	19,594	4,436 (22.6)	424 (326 - 470)	—
3 doses 7–119 days earlier	1,539	175 (11.4)	77 (46 - 100)	49 (39 - 58)
**18-49 years**				
**3 doses vs. 2 doses**				
2 doses ≥150 days earlier (Ref)	10,409	2,217 (21.3)	399 (305 - 446)	—
3 doses 7–119 days earlier	619	66 (10.7)	74 (43 - 101)	48 (32 - 60)
**50-64 years**				
**3 doses vs. 2 doses**				
2 doses ≥150 days earlier (Ref)	4,080	985 (24.1)	428 (336 - 466)	—
3 doses 7–119 days earlier	360	41 (11.4)	79 (51 - 102)	48 (27 - 63)
**4 doses vs. 3 doses**				
3 doses ≥120 days earlier (Ref)	4,978	1,069 (21.5)	220 (192 - 248)	—
4 doses 7–119 days earlier	1,452	210 (14.5)	66 (41 - 89)	23 (8 - 35)
**≥65 years**				
**3 doses vs. 2 doses**				
2 doses ≥150 days earlier (Ref)	5,105	1,234 (24.2)	469 (417 - 499)	—
3 doses 7–119 days earlier	560	68 (12.1)	77 (48 - 99)	44 (23 - 59)
**4 doses vs. 3 doses**				
3 doses ≥120 days earlier (Ref)	9,881	2,134 (21.6)	242 (212 - 268)	—

© 2023 Link-Gelles R et al. *JAMA Network Open.*

Encounter type/comparison type/age group/specific vaccination status comparison	Total	SARS-CoV-2–positive, No. (%)	Days since recent dose, median (IQR)	Adjusted relative VE[a] % (95% CI)
4 doses 7–119 days earlier	5,728	821 (14.3)	75 (50 - 93)	35 (28 - 41)

Abbreviations: CI, confidence interval; ED, emergency department; IQR, interquartile range; Ref, referent group; UC, urgent care; VE, vaccine effectiveness.

[a] An adjusted relative VE >0 indicates that COVID-19–associated ED/UC encounters were associated with being 2-dose versus 3-dose vaccinated or being 3-dose versus 4-dose vaccinated. VE estimates were adjusted for age, geographic region, calendar time (days since January 1, 2021), and local virus circulation (percentage of SARS-CoV-2–positive results from testing within the counties surrounding the facility on the date of the encounter) and weighted for inverse propensity to be 3-dose or 2-dose vaccinated or 4-dose or 3-dose vaccinated (calculated separately for each VE estimate). Generalized boosted regression trees were used to estimate the propensity to be 3-dose or 4-dose vaccinated based on the following socio-demographic, facility, and medical factors: age, sex, race, ethnicity, Medicaid status, calendar date, geographic region, local SARS-CoV-2 circulation on the day of each medical visit, urban-rural classification of facility, chronic respiratory condition, chronic non-respiratory condition, asthma, chronic obstructive pulmonary disease, other chronic lung disease, heart failure, ischemic heart disease, hypertension, other heart disease, stroke, other cerebrovascular disease, diabetes type 1, diabetes type 2, diabetes due to underlying conditions or other specified diabetes, other metabolic disease (excluding diabetes), clinical obesity, clinical underweight, renal disease, liver disease, blood disorder, dementia, other neurological/musculoskeletal disorder, Down syndrome, and the presence of at least one prior molecular or rapid antigen SARS-CoV-2 test record documented in the electronic medical record ≥15 days before the medical encounter date (pre-vaccination).

© 2023 Link-Gelles R et al. *JAMA Network Open.*

eTable 5. Relative Vaccine Effectiveness Associated With Protection Against Laboratory-Confirmed COVID-19–Associated Hospitalization of 3 vs 2 or 4 vs 3 mRNA COVID-19 Vaccine Doses, by Age Group

Encounter type/comparison type/age group/specific vaccination status comparison	Total	SARS-CoV-2–positive, No. (%)	Days since recent dose, median (IQR)	Adjusted relative VE[a] % (95% CI)
**Hospitalizations**				
**Comparisons to assess incremental benefit of additional dose when recommended**				
**All adults**				
**3 doses vs. 2 doses**				
2 doses ≥150 days earlier (Ref)	4,845	824 (17.0)	450 (373 - 491)	—
3 doses 7–119 days earlier	429	33 (7.7)	76 (43 - 100)	57 (35 - 72)
**18-49 years**				
**3 doses vs. 2 doses[b]**				
2 doses ≥150 days earlier (Ref)	887	109 (12.3)	399 (304 - 448)	—
3 doses 7–119 days earlier[c]	—	—	—	—
**50-64 years**				
**3 doses vs. 2 doses[b]**				
2 doses ≥150 days earlier (Ref)	1,130	159 (14.1)	427 (336 - 465)	—
3 doses 7–119 days earlier[c]	—	—	—	—
**4 doses vs. 3 doses[b]**				
3 doses ≥120 days earlier (Ref)	1,121	133 (11.9)	220 (194 - 248)	—
4 doses 7–119 days earlier	273	25 (9.2)	70 (43 - 89)	—
**≥65 years**				
**3 doses vs. 2 doses**				
2 doses ≥150 days earlier (Ref)	2,828	556 (19.7)	473 (422 - 503)	—
3 doses 7–119 days earlier	289	26 (9.0)	72 (42 - 98)	59 (33 - 75)
**4 doses vs. 3 doses**				
3 doses ≥120 days earlier (Ref)	4,838	913 (18.9)	240 (211 - 266)	—
4 doses 7–119 days earlier	2,179	277 (12.7)	73 (48 - 91)	37 (25 - 46)

Abbreviations: CI, confidence interval; IQR, interquartile range; Ref, referent group; VE, vaccine effectiveness.

© 2023 Link-Gelles R et al. *JAMA Network Open.*

[a] An adjusted relative VE >0 indicates that COVID-19–associated hospitalization was associated with being 2-dose versus 3-dose vaccinated or being 3-dose versus 4-dose vaccinated. VE estimates were adjusted for age, geographic region, calendar time (days since January 1, 2021), and local virus circulation (percentage of SARS-CoV-2–positive results from testing within the counties surrounding the facility on the date of the encounter) and weighted for inverse propensity to be 3-dose or 2-dose vaccinated or 4-dose or 3-dose vaccinated (calculated separately for each VE estimate). Generalized boosted regression trees were used to estimate the propensity to be 3-dose or 4-dose vaccinated based on the following socio-demographic, facility, and medical factors: age, sex, race, ethnicity, Medicaid status, calendar date, geographic region, local SARS-CoV-2 circulation on the day of each medical visit, urban-rural classification of facility, hospital type, number of hospital beds, chronic respiratory condition, chronic non-respiratory condition, asthma, chronic obstructive pulmonary disease, other chronic lung disease, heart failure, ischemic heart disease, hypertension, other heart disease, stroke, other cerebrovascular disease, diabetes type 1, diabetes type 2, diabetes due to underlying conditions or other specified diabetes, other metabolic disease (excluding diabetes), clinical obesity, clinical underweight, renal disease, liver disease, blood disorder, dementia, other neurological/musculoskeletal disorder, Down syndrome, and the presence of at least one prior molecular or rapid antigen SARS-CoV-2 test record documented in the electronic medical record ≥15 days before the medical encounter date (pre-vaccination).
[b] Adjusted VE estimates are not shown for vaccination status comparisons with confidence intervals greater than 50 percentage points around the VE estimate.
[c] In vaccination status subgroups with <10 SARS-CoV-2–positive cases, all numbers in the row were removed because of small cell sizes.

© 2023 Link-Gelles R et al. *JAMA Network Open.*

eFigure 1. Flowchart for the Selection of Emergency Department and Urgent Care Encounters

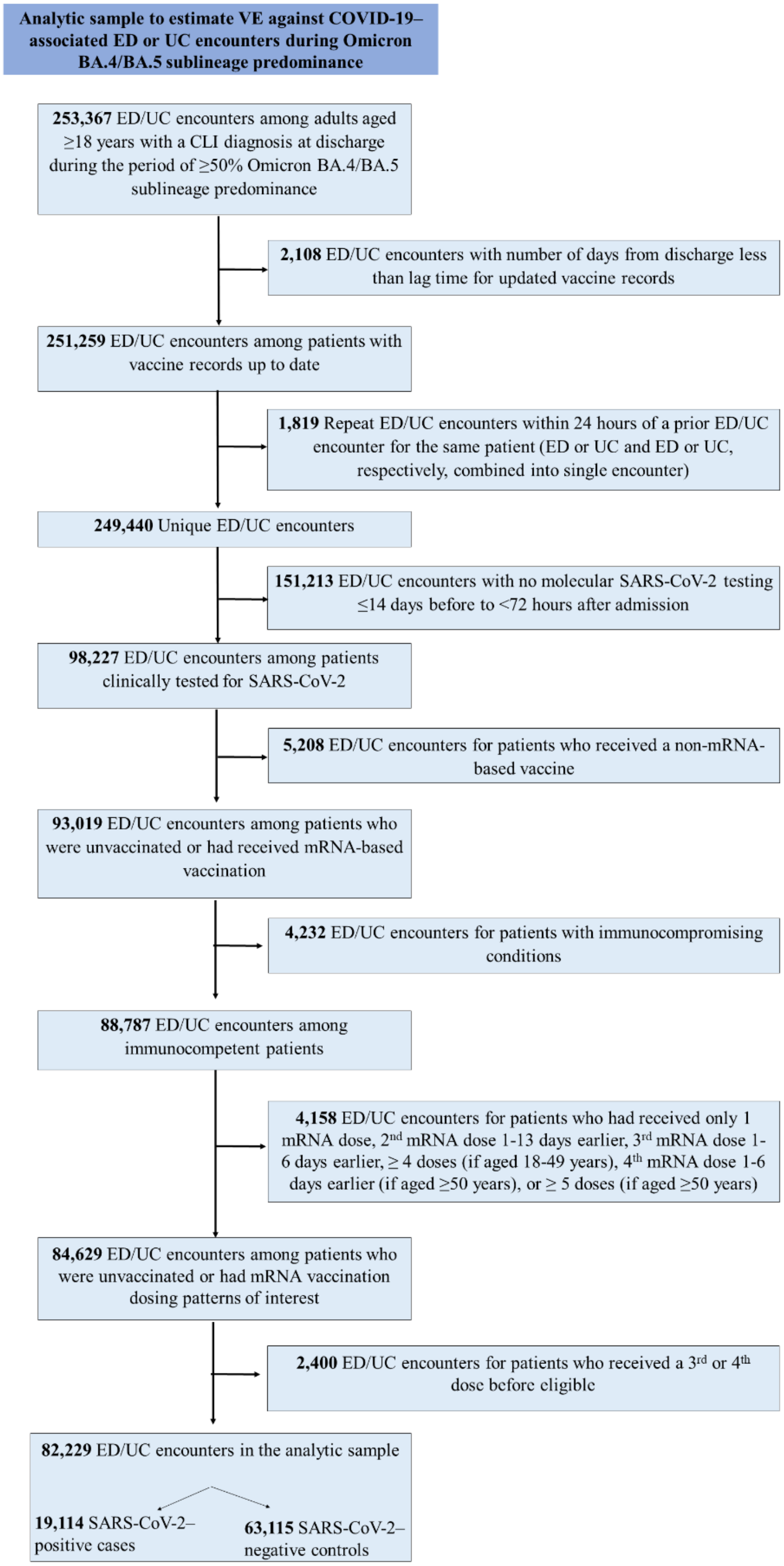

CLI indicates COVID-19–like illness; ED, emergency department; UC, urgent care; VE, vaccine effectiveness.

© 2023 Link-Gelles R et al. *JAMA Network Open.*

eFigure 2. Effectiveness Associated With mRNA COVID-19 Vaccine for Protection Against Laboratory-Confirmed COVID-19–Associated Emergency Department or Urgent Care Encounters and Hospitalization Among All Adults

Encounter type/age group/vaccination status	Total	SARS-CoV-2–positive, n (%)	Days since recent dose, median (IQR)	Adjusted VE % (95% CI)
ED or UC Encounters				
All adults				
Unvaccinated (Ref)	29,365	8,401 (28.6)		
2 doses (14-149 days earlier)	652	96 (14.7)	105 (70 - 132)	47 (33 - 58)
2 doses (150+ days earlier)	19,594	4,436 (22.6)	424 (326 - 470)	28 (24 - 31)
3 doses (7-119 days earlier)	1,539	175 (11.4)	77 (46 - 100)	62 (54 - 68)
3 doses (120+ days earlier)	23,417	4,909 (21.0)	228 (197 - 257)	32 (29 - 36)
Hospitalizations				
All adults				
Unvaccinated (Ref)	6,337	1,266 (20.0)		
2 doses (14-149 days earlier)	141	18 (12.8)	96 (61 - 120)	
2 doses (150+ days earlier)	4,845	824 (17.0)	450 (373 - 491)	25 (17 - 32)
3 doses (7-119 days earlier)	429	33 (7.7)	76 (43 - 100)	68 (50 - 80)
3 doses (120+ days earlier)	6,656	1,118 (16.8)	235 (204 - 262)	36 (29 - 42)

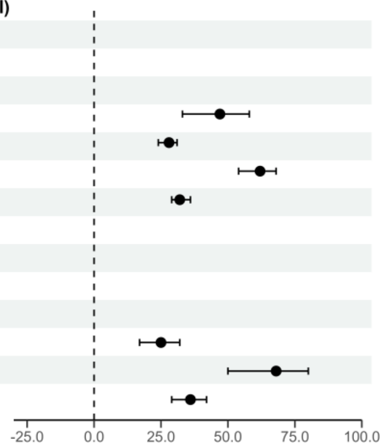

Vaccine effectiveness (VE) estimates were adjusted for age, geographic region, calendar time (days since January 1, 2021), and local virus circulation (percentage of SARS-CoV-2–positive results from testing within the counties surrounding the facility on the date of the encounter) and weighted for inverse propensity to be vaccinated or unvaccinated (calculated separately for each VE estimate). Generalized boosted regression trees were used to estimate the propensity to be vaccinated based on the following socio-demographic, facility, and medical factors: age, sex, race, ethnicity, Medicaid status, calendar date, geographic region, local SARS-CoV-2 circulation on the day of each medical visit, urban-rural classification of facility, hospital type, number of hospital beds, chronic respiratory condition, chronic non-respiratory condition, asthma, chronic obstructive pulmonary disease, other chronic lung disease, heart failure, ischemic heart disease, hypertension, other heart disease, stroke, other cerebrovascular disease, diabetes type 1, diabetes type 2, diabetes due to underlying conditions or other specified diabetes, other metabolic disease (excluding diabetes), clinical obesity, clinical underweight, renal disease, liver disease, blood disorder, dementia, other neurological/musculoskeletal disorder, Down syndrome, and the presence of at least one prior molecular or rapid antigen SARS-CoV-2 test record documented in the electronic medical record ≥15 days before the medical encounter date (pre-vaccination, if vaccinated). VE estimates are not shown for vaccination status comparisons with confidence intervals (CIs) greater than 50 percentage points around the VE estimate. ED indicates emergency department; IQR, interquartile range; Ref, referent group; UC, urgent care.

eFigure 3. Effectiveness Associated With mRNA COVID-19 Vaccine for Protection Against Laboratory-Confirmed COVID-19–Associated Intensive Care Unit Admission and/or In-Hospital Death, by Age Group

Encounter type/age group/vaccination status	Total	SARS-CoV-2–positive, n (%)	Days since recent dose, median (IQR)	Adjusted VE % (95% CI)
ICU admissions and/or in-hospital deaths				
All adults				
Unvaccinated (Ref)	5,271	200 (3.8)		
2 doses (14-149 days earlier)				
2 doses (150+ days earlier)	4,138	117 (2.8)	448 (372 - 490)	33 (15 - 48)
3 doses (7-119 days earlier)				
3 doses (120+ days earlier)	5,699	161 (2.8)	234 (203 - 262)	41 (23 - 55)
18-49 years				
Unvaccinated (Ref)	1,553	48 (3.1)		
2 doses (14-149 days earlier)				
2 doses (150+ days earlier)	797	19 (2.4)	402 (307 - 449)	
3 doses (7-119 days earlier)				
3 doses (120+ days earlier)	638	13 (2.0)	213 (186 - 242)	
50-64 years				
Unvaccinated (Ref)	1,377	39 (2.8)		
2 doses (14-149 days earlier)				
2 doses (150+ days earlier)	995	24 (2.4)	426 (335 - 464)	
3 doses (7-119 days earlier)				
3 doses (120+ days earlier)	1,006	18 (1.8)	220 (194 - 248)	
4 doses (7-59 days earlier)				
4 doses (60+ days earlier)				
65+ years				
Unvaccinated (Ref)	2,341	113 (4.8)		
2 doses (14-149 days earlier)				
2 doses (150+ days earlier)	2,346	74 (3.1)	472 (419 - 502)	44 (23 - 59)
3 doses (7-119 days earlier)				
3 doses (120+ days earlier)	4,055	130 (3.2)	240 (210 - 266)	46 (28 - 60)
4 doses (7-59 days earlier)	690	14 (2.0)	38 (24 - 50)	
4 doses (60+ days earlier)	1,364	25 (1.8)	88 (75 - 104)	62 (31 - 79)

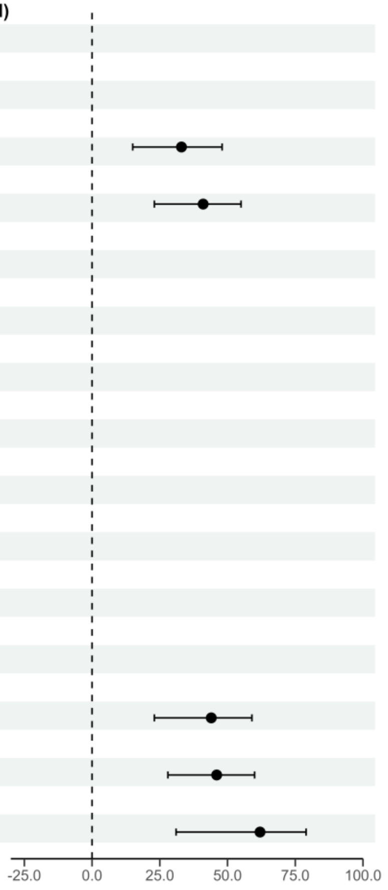

Vaccine effectiveness (VE) estimates were adjusted for age, geographic region, calendar time (days since January 1, 2021), and local virus circulation (percentage of SARS-CoV-2–positive results from testing within the counties surrounding the facility on the date of the encounter) and weighted for inverse propensity to be vaccinated or unvaccinated (calculated separately for each VE estimate). Generalized boosted regression trees were used to estimate the propensity to be vaccinated based on the following socio-demographic, facility, and medical factors: age, sex, race, ethnicity, Medicaid status, calendar date, geographic region, local SARS-CoV-2 circulation on the day of each medical visit, urban-rural classification of facility, hospital type, number of hospital beds, chronic respiratory condition, chronic non-respiratory condition, asthma, chronic obstructive pulmonary disease, other chronic lung disease, heart failure, ischemic heart disease, hypertension, other heart disease, stroke, other cerebrovascular disease, diabetes type 1, diabetes type 2, diabetes due to underlying conditions or other specified diabetes, other metabolic disease (excluding diabetes), clinical obesity, clinical underweight, renal disease, liver disease, blood disorder, dementia, other neurological/musculoskeletal disorder, Down syndrome, and the presence of at least one prior molecular or rapid antigen SARS-CoV-2 test record documented in the electronic

© 2023 Link-Gelles R et al. *JAMA Network Open.*

medical record ≥15 days before the medical encounter date (pre-vaccination, if vaccinated). In-hospital death was defined as death in the hospital occurring ≤28 days after admission. VE estimates are not shown for vaccination status comparisons with confidence intervals (CIs) greater than 50 percentage points around the VE estimate. Adjusted VE could not be calculated for the following subgroups due to lack of model convergence: 18-49 years, 3 doses (7-119 days earlier); 50-64 years, 2 doses (14-149 days earlier); and 65+ years, 2 doses (14-149 days earlier). In vaccination status subgroups with <10 SARS-CoV-2–positive cases, all numbers in the row were removed because of small cell sizes. Analyses for intensive care unit (ICU) admission and/or in-hospital death included SARS-CoV-2–positive cases with ICU admission and/or in-hospital death and all SARS-CoV-2–negative hospitalized controls. IQR indicates interquartile range; Ref, referent group.

© 2023 Link-Gelles R et al. *JAMA Network Open.*

eFigure 4. Effectiveness Associated With mRNA COVID-19 Vaccine for Protection Against Laboratory-Confirmed COVID-19–Associated Emergency Department or Urgent Care Encounters, by mRNA Vaccine Product(s) Received

Encounter type/age group/vaccination status	Total	SARS-CoV-2–positive, n (%)	Days since recent dose, median (IQR)	Adjusted VE % (95% CI)
ED or UC Encounters				
All adults, Moderna vaccines				
Unvaccinated (Ref)	29,365	8,401 (28.6)		
2 doses (14-149 days earlier)	241	36 (14.9)	99 (66 - 131)	51 (30 - 66)
2 doses (150+ days earlier)	7,329	1,599 (21.8)	437 (363 - 480)	30 (25 - 34)
3 doses (7-119 days earlier)	429	41 (9.6)	76 (46 - 100)	72 (61 - 80)
3 doses (120+ days earlier)	8,261	1,725 (20.9)	226 (198 - 250)	29 (23 - 34)
50+ years, Moderna vaccines				
Unvaccinated (Ref)	10,188	2,985 (29.3)		
2 doses (14-149 days earlier)	114	12 (10.5)	89 (60 - 119)	71 (48 - 84)
2 doses (150+ days earlier)	3,946	896 (22.7)	456 (404 - 490)	31 (24 - 37)
3 doses (7-119 days earlier)	290	31 (10.7)	78 (49 - 101)	67 (52 - 78)
3 doses (120+ days earlier)	5,613	1182 (21.1)	229 (203 - 252)	34 (28 - 40)
4 doses (7-59 days earlier)	855	83 (9.7)	39 (24 - 49)	67 (56 - 76)
4 doses (60+ days earlier)	1,792	260 (14.5)	90 (77 - 105)	49 (38 - 58)
All adults, Pfizer-BioNTech vaccines				
Unvaccinated (Ref)	29,365	8,401 (28.6)		
2 doses (14-149 days earlier)	377	60 (15.9)	112 (72 - 133)	45 (27 - 59)
2 doses (150+ days earlier)	12,177	2,821 (23.2)	413 (316 - 464)	24 (19 - 27)
3 doses (7-119 days earlier)	913	114 (12.5)	78 (48 - 101)	56 (45 - 65)
3 doses (120+ days earlier)	12,821	2,768 (21.6)	233 (198 - 266)	28 (24 - 33)
50+ years, Pfizer BioNTech vaccines				
Unvaccinated (Ref)	10,188	2,985 (29.3)		
2 doses (14-149 days earlier)	153	15 (9.8)	104 (65 - 130)	68 (45 - 81)
2 doses (150+ days earlier)	5,186	1,315 (25.4)	447 (354 - 486)	19 (12 - 25)
3 doses (7-119 days earlier)	501	64 (12.8)	79 (51 - 101)	56 (40 - 68)
3 doses (120+ days earlier)	7,998	1,796 (22.5)	244 (208 - 273)	30 (25 - 36)
4 doses (7-59 days earlier)	1,283	161 (12.6)	39 (24 - 50)	60 (50 - 68)
4 doses (60+ days earlier)	2,833	457 (16.1)	89 (76 - 105)	46 (36 - 54)
All adults, Heterologous vaccines				
Unvaccinated (Ref)	29,365	8,401 (28.6)		
2 doses (14-149 days earlier)				
2 doses (150+ days earlier)	88	16 (18.2)	259 (217 - 421)	
3 doses (7-119 days earlier)	197	20 (10.2)	75 (41 - 99)	70 (52 - 81)
3 doses (120+ days earlier)	2,335	416 (17.8)	216 (192 - 241)	38 (29 - 46)
50+ years, Heterologous vaccines				
Unvaccinated (Ref)	10,188	2,985 (29.3)		
2 doses (14-149 days earlier)				
2 doses (150+ days earlier)				
3 doses (7-119 days earlier)	129	14 (10.8)	70 (40 - 94)	69 (47 - 82)
3 doses (120+ days earlier)	1,248	225 (18.0)	221 (193 - 245)	37 (25 - 48)
4 doses (7-59 days earlier)	353	48 (13.6)	38 (24 - 49)	52 (32 - 66)
4 doses (60+ days earlier)	546	88 (16.1)	87 (74 - 102)	45 (28 - 59)

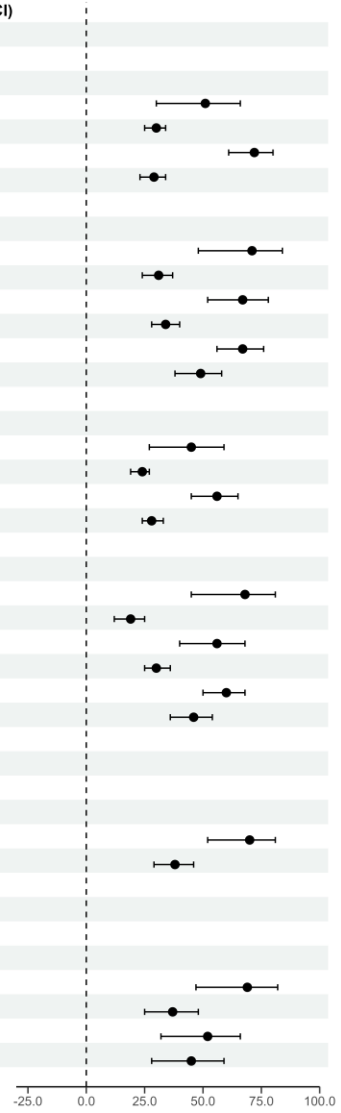

© 2023 Link-Gelles R et al. *JAMA Network Open.*

Vaccine effectiveness (VE) estimates were adjusted for age, geographic region, calendar time (days since January 1, 2021), and local virus circulation (percentage of SARS-CoV-2–positive results from testing within the counties surrounding the facility on the date of the encounter) and weighted for inverse propensity to be vaccinated or unvaccinated (calculated separately for each VE estimate). Generalized boosted regression trees were used to estimate the propensity to be vaccinated based on the following socio-demographic, facility, and medical factors: age, sex, race, ethnicity, Medicaid status, calendar date, geographic region, local SARS-CoV-2 circulation on the day of each medical visit, urban-rural classification of facility, chronic respiratory condition, chronic non-respiratory condition, asthma, chronic obstructive pulmonary disease, other chronic lung disease, heart failure, ischemic heart disease, hypertension, other heart disease, stroke, other cerebrovascular disease, diabetes type 1, diabetes type 2, diabetes due to underlying conditions or other specified diabetes, other metabolic disease (excluding diabetes), clinical obesity, clinical underweight, renal disease, liver disease, blood disorder, dementia, other neurological/musculoskeletal disorder, Down syndrome, and the presence of at least one prior molecular or rapid antigen SARS-CoV-2 test record documented in the electronic medical record ≥15 days before the medical encounter date (pre-vaccination, if vaccinated). VE estimates are not shown for vaccination status comparisons with confidence intervals (CIs) greater than 50 percentage points around the VE estimate. Adjusted VE could not be calculated for the following subgroups due to lack of model convergence: all adults, heterologous vaccines, 2 doses (14-149 days earlier); and 50+ years, heterologous vaccines, 2 doses (14-149 days earlier). In vaccination status subgroups with <10 SARS-CoV-2–positive cases, all numbers in the row were removed because of small cell sizes. ED indicates emergency department; IQR, interquartile range; Ref, referent group; UC, urgent care.

© 2023 Link-Gelles R et al. *JAMA Network Open.*

eFigure 5. Effectiveness Associated With mRNA COVID-19 Vaccine for Protection Against Laboratory-Confirmed COVID-19–Associated Hospitalization, by mRNA Vaccine Product Received

Encounter type/age group/vaccination status	Total	SARS-CoV-2–positive, n (%)	Days since recent dose, median (IQR)	Adjusted VE % (95% CI)
Hospitalizations				
All adults, Moderna vaccines				
Unvaccinated (Ref)	6,337	1,266 (20.0)		
2 doses (14-149 days earlier)				
2 doses (150+ days earlier)	2,038	337 (16.5)	458 (405 - 494)	28 (17 - 38)
3 doses (7-119 days earlier)	135	11 (8.2)	84 (49 - 103)	73 (50 - 86)
3 doses (120+ days earlier)	2,500	406 (16.2)	229 (203 - 250)	38 (27 - 46)
50+ years, Moderna vaccines				
Unvaccinated (Ref)	4,588	1,022 (22.3)		
2 doses (14-149 days earlier)				
2 doses (150+ days earlier)	1,737	303 (17.4)	464 (415 - 497)	30 (19 - 40)
3 doses (7-119 days earlier)	117	10 (8.6)	82 (49 - 100)	75 (55 - 86)
3 doses (120+ days earlier)	2,274	384 (16.9)	230 (205 - 251)	40 (30 - 48)
4 doses (7-59 days earlier)	320	25 (7.8)	38 (23 - 50)	73 (55 - 84)
4 doses (60+ days earlier)	662	74 (11.2)	88 (75 - 104)	56 (34 - 71)
All adults, Pfizer-BioNTech vaccines				
Unvaccinated (Ref)	6,337	1,266 (20.0)		
2 doses (14-149 days earlier)	77	12 (15.6)	101 (74 - 121)	
2 doses (150+ days earlier)	2,789	484 (17.4)	443 (347 - 487)	17 (7 - 27)
3 doses (7-119 days earlier)	229	22 (9.6)	74 (42 - 97)	54 (23 - 72)
3 doses (120+ days earlier)	3,606	632 (17.5)	244 (206 - 272)	33 (24 - 41)
50+ years, Pfizer-BioNTech vaccines				
Unvaccinated (Ref)	4,588	1,022 (22.3)		
2 doses (14-149 days earlier)	56	10 (17.9)	101 (76 - 120)	
2 doses (150+ days earlier)	2,207	410 (18.6)	456 (380 - 495)	20 (9 - 30)
3 doses (7-119 days earlier)	192	19 (9.9)	74 (42 - 96)	61 (31 - 78)
3 doses (120+ days earlier)	3,212	589 (18.3)	247 (211 - 274)	36 (27 - 43)
4 doses (7-59 days earlier)	424	58 (13.7)	39 (26 - 49)	44 (18 - 62)
4 doses (60+ days earlier)	889	129 (14.5)	90 (76 - 105)	53 (37 - 66)
All adults, Heterologous vaccines				
Unvaccinated (Ref)	6,337	1,266 (20.0)		
2 doses (14-149 days earlier)				
2 doses (150+ days earlier)				
3 doses (7-119 days earlier)				
3 doses (120+ days earlier)	550	80 (14.6)	221 (195 - 245)	23 (-2 - 42)
50+ years, Heterologous vaccines				
Unvaccinated (Ref)	4,588	1,022 (22.3)		
2 doses (14-149 days earlier)				
2 doses (150+ days earlier)				
3 doses (7-119 days earlier)				
3 doses (120+ days earlier)	473	73 (15.4)	222 (195 - 245)	25 (0 - 44)
4 doses (7-59 days earlier)	134	14 (10.4)	36 (21 - 49)	65 (36 - 80)
4 doses (60+ days earlier)	170	24 (14.1)	84 (72 - 101)	52 (22 - 71)

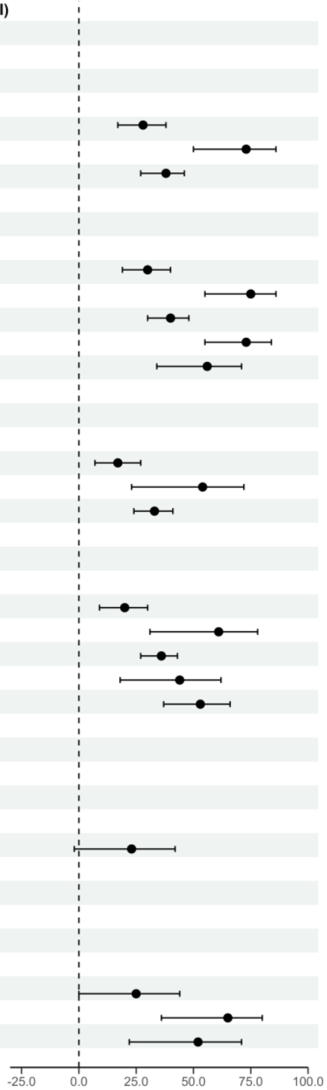

© 2023 Link-Gelles R et al. *JAMA Network Open.*

Vaccine effectiveness (VE) estimates were adjusted for age, geographic region, calendar time (days since January 1, 2021), and local virus circulation (percentage of SARS-CoV-2–positive results from testing within the counties surrounding the facility on the date of the encounter) and weighted for inverse propensity to be vaccinated or unvaccinated (calculated separately for each VE estimate). Generalized boosted regression trees were used to estimate the propensity to be vaccinated based on the following socio-demographic, facility, and medical factors: age, sex, race, ethnicity, Medicaid status, calendar date, geographic region, local SARS-CoV-2 circulation on the day of each medical visit, urban-rural classification of facility, hospital type, number of hospital beds, chronic respiratory condition, chronic non-respiratory condition, asthma, chronic obstructive pulmonary disease, other chronic lung disease, heart failure, ischemic heart disease, hypertension, other heart disease, stroke, other cerebrovascular disease, diabetes type 1, diabetes type 2, diabetes due to underlying conditions or other specified diabetes, other metabolic disease (excluding diabetes), clinical obesity, clinical underweight, renal disease, liver disease, blood disorder, dementia, other neurological/musculoskeletal disorder, Down syndrome, and the presence of at least one prior molecular or rapid antigen SARS-CoV-2 test record documented in the electronic medical record ≥15 days before the medical encounter date (pre-vaccination, if vaccinated). VE estimates are not shown for vaccination status comparisons with confidence intervals (CIs) greater than 50 percentage points around the VE estimate. Adjusted VE could not be calculated for the following subgroups due to lack of model convergence: all adults, heterologous vaccines, 2 doses (14-149 days earlier); all adults, heterologous vaccines, 3 doses (7-119 days earlier); 50+ years, heterologous vaccines, 2 doses (14-149 days earlier); and 50+ years, heterologous vaccines, 3 doses (7-119 days earlier). In vaccination status subgroups with <10 SARS-CoV-2–positive cases, all numbers in the row were removed because of small cell sizes. IQR indicates interquartile range; Ref, referent group.

© 2023 Link-Gelles R et al. *JAMA Network Open.*

eFigure 6. Effectiveness Associated With mRNA COVID-19 Vaccine for Protection Against Laboratory-Confirmed COVID-19–Associated Intensive Care Unit Admission and/or In-Hospital Death, by mRNA Vaccine Product Received

Encounter type/age group/vaccination status	Total	SARS-CoV-2–positive, n (%)	Days since recent dose, median (IQR)	Adjusted VE % (95% CI)
ICU Admissions and/or in-hospital deaths				
All adults, Moderna vaccines				
Unvaccinated (Ref)	5,271	200 (3.8)		
2 doses (14-149 days earlier)				
2 doses (150+ days earlier)	1,746	45 (2.6)	458 (405 - 493)	40 (13 - 58)
3 doses (7-119 days earlier)				
3 doses (120+ days earlier)	2,139	45 (2.1)	228 (202 - 250)	62 (44 - 75)
50+ years, Moderna vaccines				
Unvaccinated (Ref)	3,718	152 (4.1)		
2 doses (14-149 days earlier)				
2 doses (150+ days earlier)	1,473	39 (2.6)	463 (413 - 497)	40 (11 - 59)
3 doses (7-119 days earlier)				
3 doses (120+ days earlier)	1,930	40 (2.1)	230 (204 - 251)	64 (46 - 77)
4 doses (7-59 days earlier)				
4 doses (60+ days earlier)				
All adults, Pfizer-BioNTech vaccines				
Unvaccinated (Ref)	5,271	200 (3.8)		
2 doses (14-149 days earlier)				
2 doses (150+ days earlier)	2,376	71 (3)	439 (343 - 485)	23 (-3 - 42)
3 doses (7-119 days earlier)				
3 doses (120+ days earlier)	3,073	99 (3.2)	243 (205 - 272)	29 (4 - 48)
50+ years, Pfizer-BioNTech vaccines				
Unvaccinated (Ref)	3,718	152 (4.1)		
2 doses (14-149 days earlier)				
2 doses (150+ days earlier)	1,855	58 (3.1)	454 (378 - 494)	28 (0 - 48)
3 doses (7-119 days earlier)				
3 doses (120+ days earlier)	2,717	94 (3.5)	247 (209 - 273)	32 (7 - 50)
4 doses (7-59 days earlier)				
4 doses (60+ days earlier)	773	13 (1.7)	89 (76 - 105)	69 (36 - 85)
All adults, Heterologous vaccines				
Unvaccinated (Ref)	5,271	200 (3.8)		
2 doses (14-149 days earlier)				
2 doses (150+ days earlier)				
3 doses (7-119 days earlier)				
3 doses (120+ days earlier)	487	17 (3.5)	221 (196 - 244)	
50+ years, Heterologous vaccines				
Unvaccinated (Ref)	3,718	152 (4.1)		
2 doses (14-149 days earlier)				
2 doses (150+ days earlier)				
3 doses (7-119 days earlier)				
3 doses (120+ days earlier)	414	14 (3.4)	222 (195 - 245)	
4 doses (7-59 days earlier)				
4 doses (60+ days earlier)				

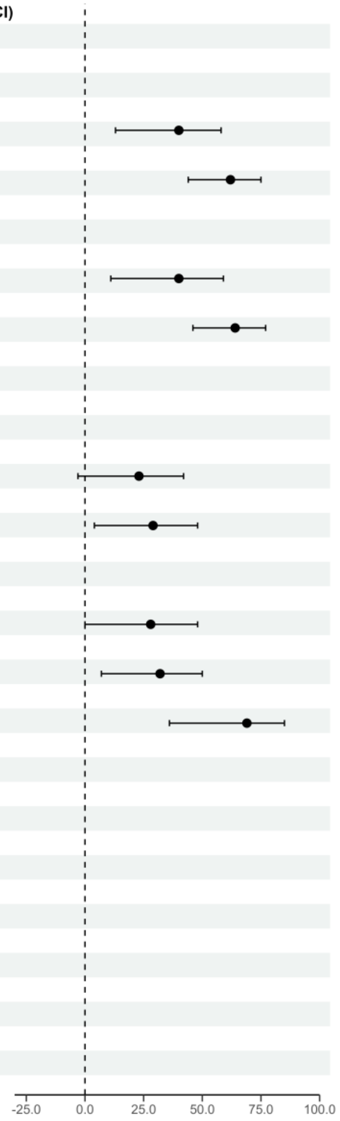

© 2023 Link-Gelles R et al. *JAMA Network Open.*

Vaccine effectiveness (VE) estimates were adjusted for age, geographic region, calendar time (days since January 1, 2021), and local virus circulation (percentage of SARS-CoV-2–positive results from testing within the counties surrounding the facility on the date of the encounter) and weighted for inverse propensity to be vaccinated or unvaccinated (calculated separately for each VE estimate). Generalized boosted regression trees were used to estimate the propensity to be vaccinated based on the following socio-demographic, facility, and medical factors: age, sex, race, ethnicity, Medicaid status, calendar date, geographic region, local SARS-CoV-2 circulation on the day of each medical visit, urban-rural classification of facility, hospital type, number of hospital beds, chronic respiratory condition, chronic non-respiratory condition, asthma, chronic obstructive pulmonary disease, other chronic lung disease, heart failure, ischemic heart disease, hypertension, other heart disease, stroke, other cerebrovascular disease, diabetes type 1, diabetes type 2, diabetes due to underlying conditions or other specified diabetes, other metabolic disease (excluding diabetes), clinical obesity, clinical underweight, renal disease, liver disease, blood disorder, dementia, other neurological/musculoskeletal disorder, Down syndrome, and the presence of at least one prior molecular or rapid antigen SARS-CoV-2 test record documented in the electronic medical record ≥15 days before the medical encounter date (pre-vaccination, if vaccinated). VE estimates are not shown for vaccination status comparisons with confidence intervals (CIs) greater than 50 percentage points around the VE estimate. Adjusted VE could not be calculated for the following subgroups due to lack of model convergence: 50+ years, Moderna vaccines, 2 doses (14-149 days earlier); 50+ years, Pfizer-BioNTech vaccines, 2 doses (14-149 days earlier); all adults, heterologous vaccines, 2 doses (14-149 days earlier); all adults, heterologous vaccines, 3 doses (7-119 days earlier); 50+ years, heterologous vaccines, 2 doses (14-149 days earlier); and 50+ years, heterologous vaccines, 3 doses (7-119 days earlier). In vaccination status subgroups with <10 SARS-CoV-2–positive cases, all numbers in the row were removed because of small cell sizes. In-hospital death was defined as death in the hospital occurring ≤28 days after admission. Analyses for intensive care unit (ICU) admission and/or in-hospital death included SARS-CoV-2–positive cases with ICU admission and/or in-hospital death and all SARS-CoV-2–negative hospitalized controls. IQR indicates interquartile range; Ref, referent group.

© 2023 Link-Gelles R et al. *JAMA Network Open.*

eFigure 7. Effectiveness Associated With mRNA COVID-19 Vaccine for Protection Against Laboratory-Confirmed COVID-19–Associated Emergency Department or Urgent Care Encounters, by Age Group, Among Patients Without a Prior Documented SARS-CoV-2 Infection

Encounter type/age group/vaccination status	Total	SARS-CoV-2–positive, n (%)	Days since recent dose, median (IQR)	Adjusted VE % (95% CI)
ED or UC Encounters, no documented prior infection				
All adults				
Unvaccinated (Ref)	23,586	7,184 (30.5)		
2 doses (14-149 days earlier)	466	74 (15.9)	102 (66 - 131)	44 (27 - 57)
2 doses (150+ days earlier)	15,398	3,870 (25.1)	426 (330 - 471)	22 (18 - 26)
3 doses (7-119 days earlier)	1,168	150 (12.8)	77 (46 - 100)	58 (49 - 66)
3 doses (120+ days earlier)	19,862	4,455 (22.4)	229 (199 - 258)	31 (27 - 34)
18-49 years				
Unvaccinated (Ref)	15,263	4,583 (30.0)		
2 doses (14-149 days earlier)	247	51 (20.6)	121 (75 - 136)	28 (1 - 48)
2 doses (150+ days earlier)	7,937	1,881 (23.7)	402 (308 - 447)	22 (17 - 27)
3 doses (7-119 days earlier)	446	55 (12.3)	73 (43 - 101)	56 (40 - 67)
3 doses (120+ days earlier)	7,070	1,549 (21.9)	217 (190 - 245)	23 (16 - 29)
50-64 years				
Unvaccinated (Ref)	4,086	1,262 (30.9)		
2 doses (14-149 days earlier)	82	12 (14.6)	100 (64 - 125)	
2 doses (150+ days earlier)	3,221	868 (26.9)	429 (342 - 467)	17 (8 - 26)
3 doses (7-119 days earlier)	278	35 (12.6)	76 (46 - 101)	62 (42 - 75)
3 doses (120+ days earlier)	4,197	969 (23.1)	221 (194 - 248)	28 (20 - 36)
4 doses (7-59 days earlier)	532	70 (13.2)	36 (22 - 48)	55 (37 - 67)
4 doses (60+ days earlier)	401	70 (17.5)	98 (79 - 114)	
65+ years				
Unvaccinated (Ref)	4,237	1,339 (31.6)		
2 doses (14-149 days earlier)	137	11 (8.0)	87 (59 - 111)	82 (66 - 91)
2 doses (150+ days earlier)	4,240	1,121 (26.4)	470 (418 - 500)	24 (17 - 31)
3 doses (7-119 days earlier)	444	60 (13.5)	80 (50 - 100)	52 (33 - 66)
3 doses (120+ days earlier)	8,595	1,937 (22.5)	243 (213 - 268)	38 (32 - 44)
4 doses (7-59 days earlier)	1,679	196 (11.7)	39 (25 - 50)	68 (60 - 74)
4 doses (60+ days earlier)	3,885	617 (15.9)	89 (77 - 105)	53 (46 - 60)

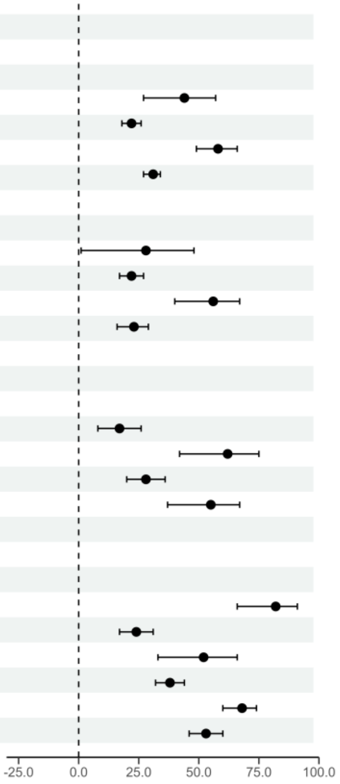

Patients included had no prior positive molecular or rapid antigen SARS-CoV-2 test result documented in the electronic medical record ≥15 days prior to the ED or UC encounter date. Vaccine effectiveness (VE) estimates were adjusted for age, geographic region, calendar time (days since January 1, 2021), and local virus circulation (percentage of SARS-CoV-2–positive results from testing within the counties surrounding the facility on the date of the encounter) and weighted for inverse propensity to be vaccinated or unvaccinated (calculated separately for each VE estimate). Generalized boosted regression trees were used to estimate the propensity to be vaccinated based on the following socio-demographic, facility, and medical factors: age, sex, race, ethnicity, Medicaid status, calendar date, geographic region, local SARS-CoV-2 circulation on the day of each medical visit, urban-rural classification of facility, chronic respiratory condition, chronic non-respiratory condition, asthma, chronic obstructive pulmonary disease, other chronic lung disease, heart failure, ischemic heart disease, hypertension, other heart disease, stroke, other cerebrovascular disease, diabetes type 1, diabetes type 2, diabetes due to underlying conditions or other specified diabetes, other metabolic disease (excluding diabetes), clinical obesity, clinical underweight, renal disease, liver disease, blood disorder, dementia, other neurological/musculoskeletal disorder, Down syndrome, and the presence of at least one prior molecular or rapid antigen SARS-CoV-2 test record documented in the electronic medical record ≥15 days before the medical encounter date (pre-vaccination, if vaccinated). VE estimates are not shown for vaccination status comparisons with confidence intervals (CIs) greater than 50 percentage points around the VE estimate. ED indicates emergency department; IQR, interquartile range; Ref, referent group; UC, urgent care.

© 2023 Link-Gelles R et al. *JAMA Network Open.*

eFigure 8. Effectiveness Associated With mRNA COVID-19 Vaccine for Protection Against Laboratory-Confirmed COVID-19–Associated Hospitalization, by Age Group, Among Patients Without a Prior Documented SARS-CoV-2 Infection

Encounter type/age group/vaccination status	Total	SARS-CoV-2–positive, n (%)	Days since recent dose, median (IQR)	Adjusted VE % (95% CI)
Hospitalizations, no documented prior infection				
All adults				
Unvaccinated (Ref)	5,290	1,139 (21.5)		
2 doses (14-149 days earlier)	110	16 (14.6)	93 (59 - 119)	
2 doses (150+ days earlier)	3,926	749 (19.1)	451 (375 - 491)	22 (12 - 30)
3 doses (7-119 days earlier)	328	28 (8.5)	76 (44 - 100)	66 (45 - 80)
3 doses (120+ days earlier)	5,735	1,010 (17.6)	235 (205 - 263)	38 (31 - 45)
18-49 years				
Unvaccinated (Ref)	1,441	209 (14.5)		
2 doses (14-149 days earlier)				
2 doses (150+ days earlier)	686	94 (13.7)	402 (306 - 449)	
3 doses (7-119 days earlier)				
3 doses (120+ days earlier)	572	62 (10.8)	213 (188 - 244)	
50-64 years				
Unvaccinated (Ref)	1,330	252 (18.9)		
2 doses (14-149 days earlier)				
2 doses (150+ days earlier)	902	141 (15.6)	425 (333 - 465)	19 (-2 - 36)
3 doses (7-119 days earlier)				
3 doses (120+ days earlier)	962	124 (12.9)	220 (194 - 248)	42 (25 - 55)
4 doses (7-59 days earlier)				
4 doses (60+ days earlier)	84	10 (11.9)	95 (83 - 111)	
65+ years				
Unvaccinated (Ref)	2,519	678 (26.9)		
2 doses (14-149 days earlier)	64	12 (18.8)	77 (57 - 101)	
2 doses (150+ days earlier)	2,338	514 (22)	473 (422 - 503)	28 (18 - 37)
3 doses (7-119 days earlier)	224	23 (10.3)	75 (44 - 100)	70 (48 - 83)
3 doses (120+ days earlier)	4,201	824 (19.6)	241 (212 - 267)	42 (33 - 49)
4 doses (7-59 days earlier)	671	82 (12.2)	38 (24 - 50)	66 (52 - 75)
4 doses (60+ days earlier)	1,447	197 (13.6)	88 (76 - 105)	61 (50 - 70)

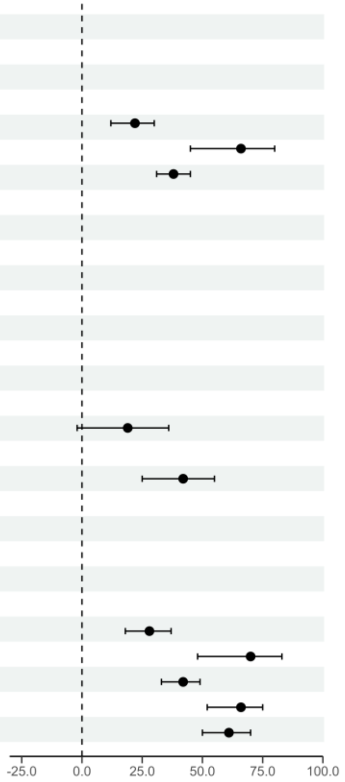

Patients included had no prior positive molecular or rapid antigen SARS-CoV-2 test result documented in the electronic medical record ≥15 days prior to the hospital admission date. Vaccine effectiveness (VE) estimates were adjusted for age, geographic region, calendar time (days since January 1, 2021), and local virus circulation (percentage of SARS-CoV-2–positive results from testing within the counties surrounding the facility on the date of the encounter) and weighted for inverse propensity to be vaccinated or unvaccinated (calculated separately for each VE estimate). Generalized boosted regression trees were used to estimate the propensity to be vaccinated based on the following socio-demographic, facility, and medical factors: age, sex, race, ethnicity, Medicaid status, calendar date, geographic region, local SARS-CoV-2 circulation on the day of each medical visit, urban-rural classification of facility, hospital type, number of hospital beds, chronic respiratory condition, chronic non-respiratory condition, asthma, chronic obstructive pulmonary disease, other chronic lung disease, heart failure, ischemic heart disease, hypertension, other heart disease, stroke, other cerebrovascular disease, diabetes type 1, diabetes type 2, diabetes due to underlying conditions or other specified diabetes, other metabolic disease (excluding diabetes), clinical obesity, clinical underweight, renal disease, liver disease, blood disorder, dementia, other neurological/musculoskeletal disorder, Down syndrome, and the presence of at least one prior molecular or rapid antigen SARS-CoV-2 test record documented in the electronic medical record ≥15 days before the medical encounter date (pre-vaccination, if vaccinated). VE estimates are not shown for vaccination status comparisons with confidence intervals (CIs) greater than 50 percentage points around the VE estimate. Adjusted VE could not be calculated for the following subgroup due to lack of model convergence: 50-64 years, 2 doses (14-149 days earlier). In vaccination status subgroups with <10 SARS-CoV-2–positive cases, all numbers in the row were removed because of small cell sizes. IQR indicates interquartile range; Ref, referent group.

© 2023 Link-Gelles R et al. *JAMA Network Open.*

eFigure 9. Effectiveness Associated With mRNA COVID-19 Vaccine for Protection Against Laboratory-Confirmed COVID-19–Associated Intensive Care Unit Admission and/or In-Hospital Death, by Age Group, Among Patients Without a Prior Documented SARS-CoV-2 Infection

Encounter type/age group/vaccination status	Total	SARS-CoV-2–positive, n (%)	Days since recent dose, median (IQR)	Adjusted VE % (95% CI)
ICU and/or in-hospital deaths, no documented prior infection				
All adults				
Unvaccinated (Ref)	4,338	187 (4.3)		
2 doses (14-149 days earlier)				
2 doses (150+ days earlier)	3,278	101 (3.1)	449 (372 - 490)	32 (12 - 47)
3 doses (7-119 days earlier)				
3 doses (120+ days earlier)	4,866	141 (2.9)	234 (204 - 262)	41 (23 - 55)
18-49 years				
Unvaccinated (Ref)	1,275	43 (3.4)		
2 doses (14-149 days earlier)				
2 doses (150+ days earlier)	607	15 (2.5)	404 (309 - 449)	
3 doses (7-119 days earlier)				
3 doses (120+ days earlier)	522	12 (2.3)	213 (187 - 243)	
50-64 years				
Unvaccinated (Ref)	1,116	38 (3.4)		
2 doses (14-149 days earlier)				
2 doses (150+ days earlier)	780	19 (2.4)	424 (332 - 462)	
3 doses (7-119 days earlier)				
3 doses (120+ days earlier)	854	16 (1.9)	220 (194 - 248)	
4 doses (7-59 days earlier)				
4 doses (60+ days earlier)				
65+ years				
Unvaccinated (Ref)	1,947	106 (5.4)		
2 doses (14-149 days earlier)				
2 doses (150+ days earlier)	1,891	67 (3.5)	473 (419 - 502)	40 (17 - 56)
3 doses (7-119 days earlier)				
3 doses (120+ days earlier)	3,490	113 (3.2)	240 (211 - 267)	48 (29 - 62)
4 doses (7-59 days earlier)	601	12 (2.0)	38 (25 - 50)	
4 doses (60+ days earlier)	1,273	23 (1.8)	88 (75 - 104)	65 (36 - 81)

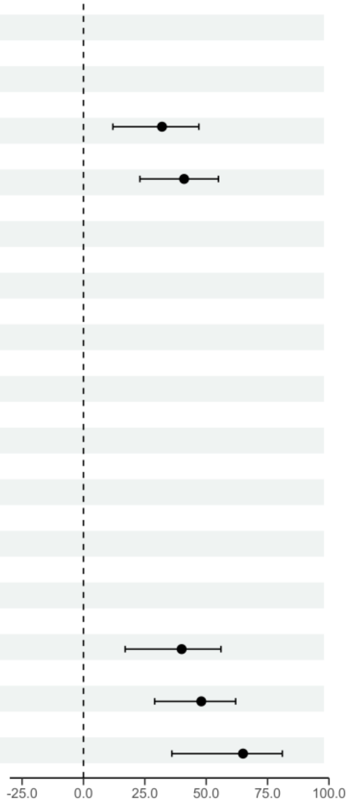

Patients included had no prior positive molecular or rapid antigen SARS-CoV-2 test result documented in the electronic medical record ≥15 days prior to the hospital admission date. Vaccine effectiveness (VE) estimates were adjusted for age, geographic region, calendar time (days since January 1, 2021), and local virus circulation (percentage of SARS-CoV-2–positive results from testing within the counties surrounding the facility on the date of the encounter) and weighted for inverse propensity to be vaccinated or unvaccinated (calculated separately for each VE estimate). Generalized boosted regression trees were used to estimate the propensity to be vaccinated based on the following socio-demographic, facility, and medical factors: age, sex, race, ethnicity, Medicaid status, calendar date, geographic region, local SARS-CoV-2 circulation on the day of each medical visit, urban-rural classification of facility, hospital type, number of hospital beds, chronic respiratory condition, chronic non-respiratory condition, asthma, chronic obstructive pulmonary disease, other chronic lung disease, heart failure, ischemic heart disease, hypertension, other heart disease, stroke, other cerebrovascular disease, diabetes type 1, diabetes type 2, diabetes due to underlying conditions or other specified diabetes, other metabolic disease (excluding diabetes), clinical obesity, clinical underweight, renal disease, liver disease, blood disorder, dementia, other neurological/musculoskeletal disorder, Down syndrome, and the presence of at least one prior molecular or rapid antigen SARS-CoV-2 test record documented in the electronic medical record ≥15 days before the medical encounter date (pre-vaccination, if vaccinated). VE estimates are not shown for vaccination status comparisons with confidence intervals (CIs) greater than 50 percentage points around the VE estimate. Adjusted VE could not be calculated for the following subgroups due to lack of model convergence: 18-49 years, 3

© 2023 Link-Gelles R et al. *JAMA Network Open.*

doses (7-119 days earlier); 50-64 years, 2 doses (14-149 days earlier); and 65+ years, 2 doses (14-149 days earlier). In vaccination status subgroups with <10 SARS-CoV-2–positive cases, all numbers in the row were removed because of small cell sizes. In-hospital death was defined as death in the hospital occurring ≤28 days after admission. Analyses for intensive care unit (ICU) admission and/or in-hospital death included SARS-CoV-2–positive cases with ICU admission and/or in-hospital death and all SARS-CoV-2–negative hospitalized controls. IQR indicates interquartile range; Ref, referent group.

© 2023 Link-Gelles R et al. *JAMA Network Open.*
