# Supplementary material for: Estimation of COVID-19 mRNA Vaccine Effectiveness and COVID-19 Illness and Severity by Vaccination Status During Omicron BA.4 and BA.5 Sublineage Periods
Source: JAMA Netw Open. 2023 Mar 15;6(3):e232598. doi: 10.1001/jamanetworkopen.2023.2598 (PMC10018321; doi:10.1001/jamanetworkopen.2023.2598)
Supplement: Supplement 2. — Data Sharing Statement [file jamanetwopen-e232598-s002.pdf]

Data Sharing Statement

Link-Gelles. Estimation of COVID-19 mRNA Vaccine Effectiveness and COVID-19 Illness and Severity by Vaccination Status During Omicron BA.4 and BA.5 Sublineage Periods. *JAMA Netw Open*. Published March 15, 2023. doi:10.1001/jamanetworkopen.2023.2598

Data
Data available: No
